# Self-reported awareness of the prevention and early detection of oral cancer: a survey of 50+ year old people in Germany

**DOI:** 10.1007/s00432-026-06491-z

**Published:** 2026-05-18

**Authors:** Lisa Felgendreff, Eva Baumann, Sarah Habig, Rieke Scharbrodt, Michael Kalab, Astrid Dempfle, Katrin Hertrampf

**Affiliations:** 1 Department of Journalism and Communication Research, Hanover University of Music, Drama, and Media, Expo Plaza 12, Hannover, 30539 Germany; 2 Department of Oral and Maxillofacial Surgery, University Hospital Schleswig- Holstein, Campus Kiel, Arnold-Heller-Str. 3, Building B, 24105 Kiel, Germany; 3 Institute of Medical Informatics and Statistics, Kiel University, University Hospital Schleswig-Holstein, Campus Kiel, Brunswiker Str. 10, 24105 Kiel, Germany

**Keywords:** Oral cancer, Public health, Survey, Knowledge, Socioeconomic status, Early detection

## Abstract

**Purpose:**

Tumours of the oral cavity are an under-recognised type of cancer, with many people being diagnosed at a late tumour stage. This study investigated knowledge of signs, symptoms and risk factors of oral cancer in association with socio-demographic factors in a German sample, with the aim of developing an awareness campaign.

**Methods:**

Computer-assisted telephone interviews (*n* = 1801) were conducted among a representative sample of the German population aged 50 and older. Descriptive statistics of demographic variables and responses to the questionnaires were reported by means of counts and percentages. Associations between socio-demographic factors and knowledge of diagnostic items and risk factors were analysed.

**Results:**

Overall, diagnostic knowledge and knowledge of risk factors were low to moderate. The knowledge levels were even lower among participants who were older, had lower school education, or had lower net monthly household income. Although the respective vulnerable groups were well aware of tobacco consumption as a risk factor, the awareness of the risk factors of older age and alcohol consumption was lower.

**Conclusion:**

This national survey demonstrates that the German population is not sufficiently informed about the signs, symptoms and risk factors of oral cancer. Knowledge deficits were particularly associated with older age, lower levels of education and low income. The planned national awareness campaign aims to inform the public, especially vulnerable subgroups, about oral cancer, its diagnostic signs andspecific risk factors. In the development, implementation and evaluation of this campaign, age, education level and income should be considered.

**Supplementary Information:**

The online version contains supplementary material available at 10.1007/s00432-026-06491-z.

## Introduction

Tumours of the oral cavity are an under-recognised type of cancer by the general public. The annual incidence worldwide is estimated at over 389,000 cases. Also, significantly more men than women are affected: about 268,600 men vs. 120,500 women (Globocan 2022). In Europe, approximately 62,120 cases were reported in 2022, also mostly among men (41,589 men vs. 20,484 women) (Globocan 2022). In 2023 in Germany, nearly 13,000 cases were diagnosed in the oral cavity and pharynx combined (8650 men vs. 4230 women), with almost 40% localised exclusively to the oral cavity (Robert Koch Institut 2025). Although the relative 5-year survival rate has improved slightly in recent years, it is only 54% for men and 63% for women (Robert Koch Institut 2025). The majority of affected people in Germany are aged 60 and older (Hertrampf et al. 2020).

However, an earlier diagnosis would improve the prognosis and, therefore, the survival rate of those affected (Sankaranarayanan et al. 2013; Cheung et al. 2021). An effective early detection measure is to screen the oral cavity for precursor lesions and tumours as part of a standardised oral mucosal examination. This examination is painless, can be carried out in a short time and has no side effects. In Germany, it is integrated into the six-monthly or annual free dental check-up. Nevertheless, more than half of those affected by oral cancer are still diagnosed at a late stage (58% UICC: 53% women vs. 62% men) (Robert Koch Institut 2025). Our previous study, as well as international studies, has shown a lack of public knowledge of oral tumours and the associated preventive examination option. In addition, there was insufficient knowledge of the main risk factors of tobacco and alcohol consumption (Hertrampf et al. 2012; Monteiro et al. 2016; Azimi et al. 2019; Kawecki et al. 2019; Grossmann et al. 2021; Zhou et al. 2022), which are also major risk factors for cardiovascular disease, various other cancers, and numerous chronic diseases (Robert Koch Institut 2025). As dental and medical professionals can only examine and inform people who come to their practice or clinic, informing the public or target group about this type of tumour and highlighting prevention options is imperative.

In the German federal state of Schleswig-Holstein, a third-party-funded comprehensive educational campaign with scientific evaluation was carried out as a model project for Germany (Baumann et al. 2019, 2023). This model project, as well as campaigns with accompanying evaluations in the US and England, showed that a structured awareness campaign improved perception of oral cancer (Eadie et al. 2009; Watson et al. 2009; Jedele and Ismail 2010; Baumann et al. 2023). As complex, multi-level prevention strategies need to be planned and implemented in a context-sensitive manner (Michie et al. 2008; Sallis et al. 2008; Bartholomew and Mullen 2011), regional results cannot be accurately generalised to provide the national picture. Therefore, insight into the German public’s knowledge of oral cancer is needed.

Consequently, this study aimed to assess the level of knowledge of the signs, symptoms, and risk factors of oral cancer in a representative sample of the German population aged 50 years and older. The level of knowledge was explored in demographic and socioeconomic target groups, and risk habits, namely tobacco and alcohol consumption, were considered. This dataset also serves as a baseline survey and contributes to conceptualising a national awareness campaign.

## Materials and methods

The study was conducted as a representative cross-sectional survey to assess the public’s self-reported knowledge of the signs, symptoms and risk factors of oral cavity tumours. The data collection was preregistered at the platform ‘aspredicted’ (#154485, https://aspredicted.org/9j8h-3bxz.pdf).

### Study population

The study population consisted of a nationally representative sample of 1801 German residents aged 50 and older, selected according to gender and age. The sample size was based on the 31 December 2022 update of the 2011 census. At that time, 83.1 million people were living in Germany, of whom 37.4 million were aged 50 and older (Destatis 2025). The sample was representative of age groups (50–59, 60–69, 70 and older) and gender (male, female). In addition, each German federal state was included proportionally according to its size. The survey included German-speaking people aged 50 and older living in private households who able to complete it in German.

### Sampling

The computer-assisted telephone interviews (CATI) were conducted by the German polling firm the forsa Institute for Social Research and Statistical Analysis (forsa.). The company programmed the telephone version and conducted a pretest with a random sample of 50 adults. In January 2024, forsa. collected the data for the representative national survey.

The company used a multi-stage random sampling frame based on the telephone master sample of the ADM (Verband Deutscher Markt- und Sozialforscher e.V.), containing randomly generated landline and mobile phone numbers (70:30) (Forsa 2021).

A master sample was generated using the Gabler–Haeder method. A three-stage stratified random sampling procedure for landline numbers makes it possible to include households with unlisted telephone numbers in the sample, as the cluster structure of German telephone numbers is considered: (1) the area code at the beginning, followed by blocks of numbers for (2) cities and (3) districts. The last two digits are omitted and are generated randomly. Mobile phone numbers are computer-generated number sequences based on the number blocks assigned by the Federal Network Agency. Mobile phone numbers are selected at random because regional stratification is not possible within this selection frame. Overall, using the ADM telephone sample is more effective and consistent thanrandom-number sampling methods.

### Survey

The questionnaire comprised four sections; this manuscript reports on the public’s knowledge of oral cancer. For transparency, the complete questionnaire is available as Supplementary Information (SI) File 1.

The first section assessed the awareness of oral cancer (one item) (Baumann et al. 2023) and the subjective knowledge (two items) (Kahlor 2010), and tested the level of objective knowledge of these specific tumours – the signs, symptoms and possible risk factors (18 items). The original knowledge questionnaire was developed and validated by Yellowitz and Goodman in the USA (Yellowitz and Goodman 1995). The current study used a validated version that had already been successfully employed in previous studies (Hertrampf et al. 2009, 2012).

Objective knowledge was tested with five single-choice questions and one multiple-choice question on diagnostics and risk factors. In a battery of 12 items, participants were asked to identify which of the named factors increase the risk of developing oral cancer (Yes/No/I don’t know; six risk factors and six distractors). The Yellowitz and Goodman diagnostic item about lymph nodes was not assessed because it requires in-depth medical knowledge (Yellowitz and Goodman 1995).

The second section dealt with the role of dentists and oral health behaviour. Six items covered topics on visits to the dentist, health history, and communication with the dentist. Three further items asked questions about the oral mucosa examination.

The third section focused on risk perception and information behaviour. Two items assessed the perceived probability and severity of getting oral cancer in the future (Hovick et al. 2014). The participants rated their information motivation regarding oral cancer using six items (Howell and Shepperd 2016). Furthermore, participants were asked to state which group of people would be the most important one for them to talk to in the event of an oral cancer diagnosis. Another item assessed whether the media should report more often on oral cancer.

The last section gathered information on the participant. It started with the occurrence of cancer in the respondent or their close social circle (two items), the individual’s general health behaviour (seven items), tobacco consumption (four items), alcohol consumption (one item) and socio-demographic variables (age, gender, marital status, highest level of school education, net monthly household income and employment; one item each). The telephone dataset provided the state and city size.

### Statistical analysis

Data were weighed by gender and age group. Descriptive statistics of respondents’ socio-demographic characteristics and knowledge test responses were reported using weighted counts and percentages.

The responses to age, school education, net monthly household income and tobacco consumption were collapsed into categories for the analyses. Age was categorised into the age groups ‘50–59’, ‘60–69’, ‘70–79’, and ‘80 and older’ to get deeper insights into the relevance of higher age for the targeted outcomes. This difference regarding the sample quotas (i.e. ‘70 and older’) led to only slightly different proportions for these age groups compared to the update of the 2011 census (weighted study sample vs census: ‘70–79’ 18.0% vs 19.8%, ‘80 and older’ 17.8% vs 16.1%). The level of school education was summarised into low (secondary school qualifications), middle (secondary school certificates), advanced (university [of applied science] entrance diplomas) and high (university [of applied science] degrees). The net monthly household income was categorised into low (≤ 1500 euros), lower-middle (1501–2500 euros), middle (2501–3500 euros), upper-middle (3501–4500 euros) and high (> 4500 euros). Tobacco consumption was differentiated into ‘smoker’, ‘ex-smoker’ and ‘non-smoker/occasional’. Missing values resulted from ‘no answer’ responses for: net monthly household income (n = 199), school education (n = 33; including ‘other’ responses), tobacco consumption (n = 21), and alcohol consumption (n = 6). Age was recorded in years and categorised into age groups for sample quotas by forsa. Missing values in the age group variable (n = 6) were due to missing values in the continuous age variable for cases in the ‘70 and older’ category.

Knowledge test responses were recoded as 1 (correct answer) and 0 (incorrect or ‘I don’t know’). The points were added up into two sum scores: diagnostic knowledge (range: 0 to 6 points) and knowledge of risk factors (range: 0 to 7 points). The latter score summarised only factors associated with oral cancer, not the distractors.

The results for knowledge items and sum scores were reported for the total sample (*n* = 1.801) and stratified by the following socio-demographic factors: gender, age group, school education, net monthly household income, tobacco consumption, and alcohol consumption. All analyses were conducted using only valid responses, excluding missing values for socio-demographic characteristics. Due to rounding of weighted sample sizes, minor differences may exist between the total sample size and the sum of subcategories plus missing values.

At the item level and for socio-demographic characteristics, group comparisons were carried out for the socio-demographic characteristics using the χ^2^-test. In addition, the effect size Cramér’s *V* quantified the size of the observed group difference. With effect size, *V* ≥ 0.1 is interpreted as small, *V* ≥ 0.3 as medium and *V* ≥ 0.5 as large (Ellis 2010). At the sum score level, a *t*-test for independent samples was used to test for group differences in sex. For all other socio-demographic characteristics, an analysis of variance (ANOVA) or a Welch–ANOVA (in the case of variance heterogeneity) was conducted. The effect size Cohen’s *d* helped to classify the overall group differences into the categories of small (|*d*| = 0.2), medium (|*d*| = 0.5) and large (|*d*| = 0.8) (Ellis 2010). In the case of ANOVAs, the overall *η*² was converted to Cohen’s *d* (Lenhard and Lenhard 2022). Pairwise post hoc tests following the (Welch–) ANOVA on the item and the sum score level were calculated using the Bonferroni correction and, in the case of variance heterogeneity, the Games–Howell correction.

Given the large sample size, statistically significant findings can occur for irrelevant effects lacking practical importance (Sullivan and Feinn 2012; Lakens 2013). Therefore, only group differences showing at least a small effect according to Cramér’s *V* or Cohen’s *d* were considered.

## Results

### Study population

A total of 1801 participants completed the telephone interview. The average interview duration was 19.2 min (15.8 to 29.7 min). To complete the 1,801 interviews, a net sample of 5072 (100.0%) was contacted. A total of 1587 (31.3%) potential participants declined the invitation, and 244 (4.8%) dropped out. In 1,334 (26.3%) cases, the target person was not reached, and in 106 (2.1%) cases, an appointment was not possible within the field time. The completion rate was 35.5% (1801 interviews).

Of the analysed weighted sample, 845 (46.9%) were male, and 956 (53.1%) were female. Corresponding to the age distribution, about two-thirds (1151, 64.2%) of the participants were 50 to 69 years old.

To interpret the results, it is important to acknowledge that socio-demographic characteristics were intercorrelated to a small extent. The net monthly household income was higher for younger (*V* = 0.21, *p* < 0.001), male (*V* = 0.16, *p* < 0.001) and better-educated participants (*V* = 0.23, *p* < 0.001). In particular, the household size influenced the net monthly household income (*V* = 0.56, *p* < 0.001), with single households having less money at their disposal. Table 1 presents the main socio-demographic characteristics. A more detailed overview of all socio-demographic characteristics is provided in SI File 2, Table S1.

**Fig. 1:**
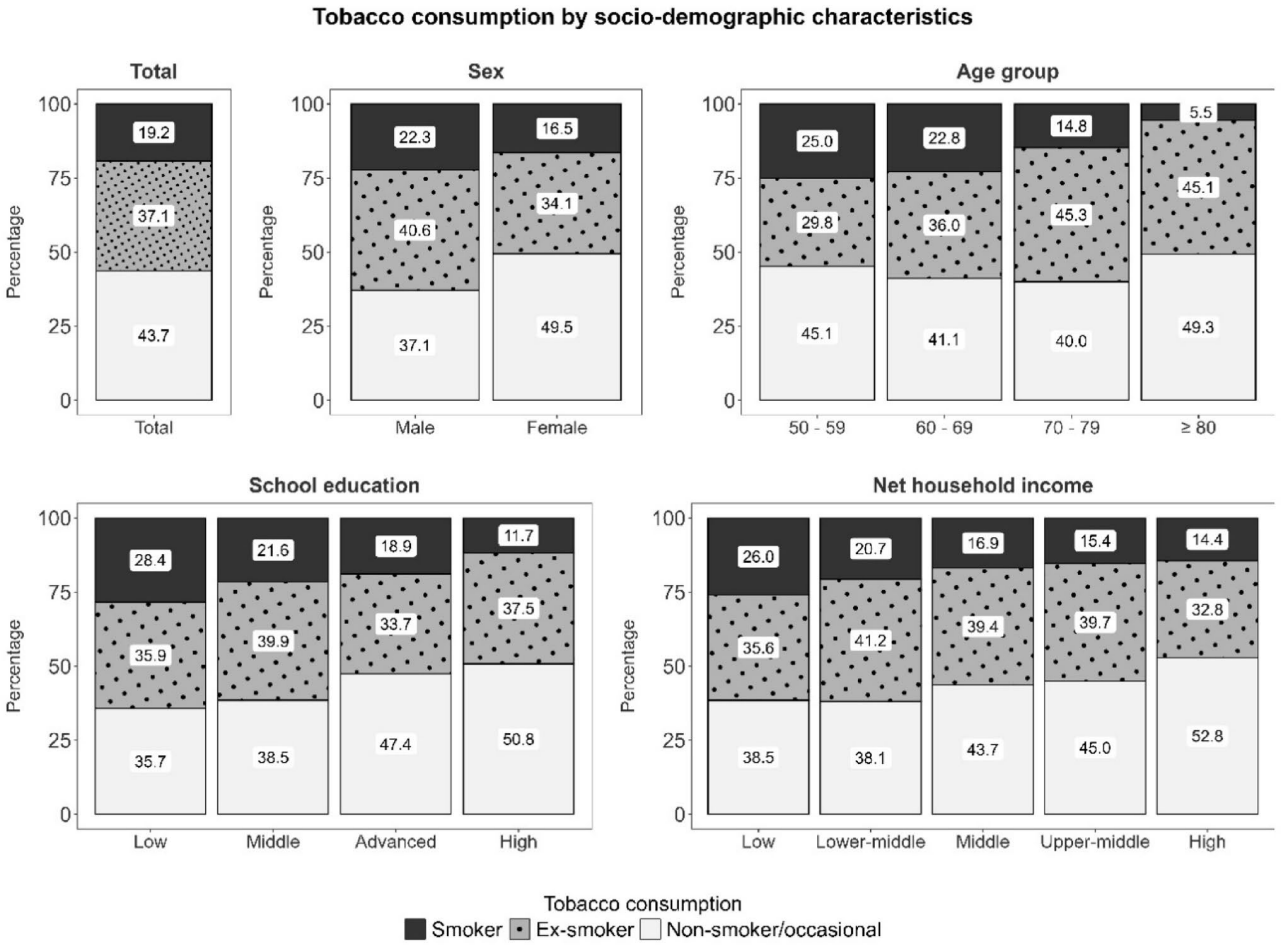
Tobacco consumption by socio-demographic characteristics

**Fig. 2:**
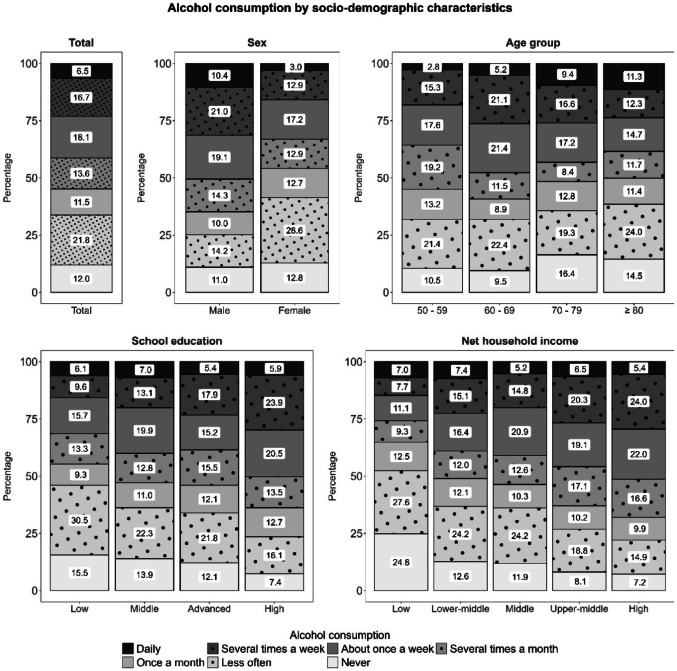
Tobacco consumption by socio-demographic characteristics

Figures 1 and 2 display how tobacco and alcohol consumption were distributed across socio-demographic characteristics. For both main risk factors – tobacco and alcohol consumption – an association with sex (*V*_*tobacco*_ = 0.13, *p*_*tobacco*_ < 0.001; *V*_*alcohol*_ = 0.24, *p*_*alcohol*_ < 0.001), age (*V*_*tobacco*_ = 0.14, *p*_*tobacco*_ < 0.001; *V*_*alcohol*_ = 0.13, *p*_*alcohol*_ < 0.001) and education (*V*_*tobacco*_ = 0.12, *p*_*tobacco*_ < 0.001; *V*_*alcohol*_ = 0.12, *p*_*alcohol*_ < 0.001) was observed (for more details, see also SI File 2, Table S1).

 Males were more likely to be current smokers (22.3% male vs. 16.5% female, *p*_*posthoc*_ = 0.002) or ex-smokers (40.6% male vs. 34.1% female, *p*_*posthoc*_ = 0.004) and were less likely to be non- or occasional smokers (37.1% male vs. 49.5% female, *p*_*posthoc*_ < 0.001) than females. Younger individuals were more likely to smoke currently (25.0% 50s vs. 22.8% 60s vs. 14.8% 70s vs. 5.5% 80+, *p*_*50vs70*_ = 0.002, *p*_*50vs80*_ < 0.001, *p*_*60vs70*_ = 0.025, *p*_*60vs80*_ < 0.001, *p*_*70vs80*_ = 0.001), whereas older individuals were more likely to be ex-smokers (29.8% 50s vs. 36.0% 60s vs. 45.3% 70s vs. 45.1% 80+, *p*_*50vs70*_ < 0.001, *p*_*50vs80*_ < 0.001, *p*_*60vs70*_ = 0.045). Moreover, participants with a lower education level (28.4% low vs. 21.6% middle vs. 18.9% advanced vs. 11.7% high, *p*_*lowVSadvanced*_ = 0.022, *p*_*lowVShigh*_ < 0.001, *p*_*middleVShigh*_ < 0.001, *p*_*advancedVShigh*_ = 0.015) were more likely to be current smokers and less likely to be non- or occasional smokers (35.7% low vs. 38.5% middle vs. 47.4% advanced vs. 50.8% high, *p*_*lowVSadvanced*_ = 0.013, *p*_*lowVShigh*_ < 0.001, *p*_*middleVShigh*_ < 0.001)

 For alcohol consumption, male participants were more likely to be drinking daily (10.4% male vs. 3.0% female, *p*_*posthoc*_ < 0.001) or several times a week (21.0% male vs. 12.9% female, *p*_*posthoc*_ < 0.001), whereas females were more likely to drink alcohol less often than once a month (14.2% male vs. 28.6% female, *p*_*posthoc*_ < 0.001). Although older participants (2.8% 50s vs. 5.2% 60s vs. 9.4% 70s vs. 11.3% 80+, *p*_*50vs70*_ < 0.001, *p*_*50vs80*_ < 0.001, *p*_*60vs80*_ = 0.007) were more likely to consume alcohol on a daily basis, those aged 60 to 69 years were most likely to consume alcohol several times a week (15.3% 50s vs. 21.1% 60s vs. 16.6% 70s vs. 12.3% 80+, *p*_*60vs80*_ = 0.007). Participants aged 50 to 59 years stated more frequently that they drink alcohol several times a month compared to the older age groups (19.2% 50s vs. 11.5% 60s vs. 8.4% 70s vs. 11.7% 80+, *p*_*50vs60*_ = 0.002, *p*_*50vs70*_ < 0.001, p_5__*0vs80*_ = 0.022). Also, those aged 70 to 79 years said more frequently that they never drink alcohol compared to participants aged 60 to 69 years (10.5% 50s vs. 9.5% 60s vs. 16.4% 70s vs. 14.5% 80+, *p*_*60vs70*_ = 0.016). Also, participants with a higher education (9.6% low vs. 13.1% middle vs. 17.9% advanced vs. 23.9% high, *p*_*lowVSadvanced*_ = 0.011, *p*_*lowVShigh*_ < 0.001, *p*_*middleVShigh*_ < 0.001) and a higher net monthly household income (overall_alcohol–income_: *V* = 0.13, *p* < .001; 7.7% low vs. 15.1% lower-middle vs. 14.8% middle vs. 20.3% upper-middle vs. 24.0% high, *p*_*lowVSupper−middle*_ = 0.002, *p*_*lowVShigh*_ < 0.001, *p*_*lower−middleVShigh*_ = 0.015, *p*_*middleVShigh*_ = 0.017) were more likely to drink alcohol several times a week. In addition, participants with a higher education and net monthly household income were less likely to drink alcohol less often than once a month (education: 30.5% low vs. 22.3% middle vs. 21.8% advanced vs. 16.1% high, *p*_*lowVShigh*_ < 0.001; net monthly household income: 27.6% low vs. 24.2% lower-middle vs. 24.2% middle vs. 18.8% upper-middle vs. 14.9% high, *p*_*lowVShigh*_ = 0.001, *p*_*lower−middleVShigh*_ = 0.007, *p*_*middleVShigh*_ = 0.011) or never (education: 15.5% low vs. 13.9% middle vs. 12.1% advanced vs. 7.4% high, *p*_*lowVShigh*_ = 0.001, *p*_*middleVShigh*_ = 0.003; net monthly household income: 24.8% low vs. 12.6% lower-middle vs. 11.9% middle vs. 8.1% upper-middle vs. 7.2% high, *p*_*lowVSlower−middle*_ = 0.002, *p*_*lowVSmiddle*_ = 0.001, *p*_*lowVSupper−middle*_ < 0.001, *p*_*lowVShigh*_ < 0.001) than those with lower educational levels. Moreover, participants with the lowest net monthly household income tended to drink alcohol once a week less often than those with a higher income (11.1% low vs. 16.4% lower-middle vs. 20.9% middle vs. 19.1% upper-middle vs. 22.0% high, *p*_*lowVSmiddle*_ = 0.036, *p*_*lowVShigh*_ = 0.010)

**Table 1:** Characteristics of study population

Characteristics	Total, *n* (%)
*Sex*	
Male	845 (46.9)
Female	956 (53.1)
*Age group*	
50–59	612 (34.1)
60–69	539 (30.1)
70–79	323 (18.0)
≥ 80	320 (17.8)
*School education*	
Low	318 (18.0)
Middle	524 (29.6)
Advanced	363 (20.6)
High	563 (31.8)
*Net monthly household income*	
Low	200 (12.5)
Lower-middle	392 (24.4)
Middle	328 (20.5)
Upper-middle	246 (15.4)
High	437 (27.3)

### Knowledge of diagnostic items

 Overall, the results for the diagnostic knowledge of the tumour, signs and symptoms demonstrated low public knowledge (Tables 2 and 3). On average, participants gave 2.67 (*SD* = 1.24) correct answers across six aspects of signs and symptoms. Less than two-thirds knew that oral cancer is often diagnosed at advanced stages (64.4%) and that the tongue is one of the most common sites (58.5%). Even fewer participants knew that the floor of the mouth is also one of the most common sites of oral cancer (33.3%). Furthermore, only around one-third knew that doctors diagnose most cases of oral cancer in the age group 60 years and above (37.1%)

 For differences in overall diagnostic knowledge based on the participants’ socio-demographic background, age group (*d* = 0.36), school education (*d* = 0.37) and net monthly household income (*d* = 0.39) showed small effects. A post hoc test with Bonferroni correction revealed that participants aged between 50 and 69 had a greater knowledge of diagnostic aspects than older participants (50s vs. 70s: *p* < 0.001, *d* = 0.39; 50s vs. ≥ 80: *p* < 0.001, *d* = 0.40; 60s vs. 70s: *p* < 0.001, *d* = 0.35; 60s vs. ≥ 80: *p* < 0.001, *d* = 0.36). For school education, participants with a low or middle education level had on average a lower diagnostic knowledge score than participants with an advanced (Bonferroni correction; low: *p* < 0.001, *d *=− 0.46; middle: *p* < 0.001, *d * =−0.39) or high education level (low: *p* < 0.001, *d * =−0.37; middle: *p* < 0.001, *d * =−0.30). Participants living in a household with a low or lower-middle net income had lower knowledge scores than households with more income (Bonferroni correction; low vs. middle: *p* < 0.001, *d *=− 0.41; low vs. upper-middle: *p* < 0.001, *d * =−0.48; low vs. high: *p* < 0.001, *d *=− 0.54; lower-middle vs. middle: *p* = 0.009, *d *=−0.25; lower-middle vs. upper-middle: *p* < 0.001, *d *=− 0.33; lower-middle vs. high: *p* < 0.001, *d * =−0.38)

 There were no differences in diagnostic knowledge with respect to tobacco and alcohol consumption habits

SI File 2, Tables S2 and S3, provide results by regionand post hoc tests of items with a relevant group difference based on the magnitude of the effect size

**Table 2:** Knowledge of diagnostic items of oral cancer by socio-demographic characteristics

Sample characteristics	The most common sites		Asympto-matic at early stages	Majority diagnosis at age 60+	Diagnose at an advanced stage	Appearance of early lesions	Sum score diagnostics
	Tongue	Floor of the mouth					
	%	%	%	%	%	%	*M* (*SD*)
Total	58.5	33.3	37.6	37.1	64.4	36.4	2.67 (1.24)
Sex, *overall statistical test*	χ^2^(1) = 3.22, *p* = 0.073, *V* = 0.04	χ^2^(1) = 0.00, *p* = 0.997, *V* = 0.00	χ^2^(1) = 0.23, *p* = 0.628, *V* = 0.01	χ^2^(1) = 9.35, *p* = 0.002, *V* = 0.07	χ^2^(1) = 0.30, *p* = 0.584, *V* = 0.01	χ^2^(1) = 4.62, *p* = 0.032, *V* = 0.05	t(1799) = 1.32, *p* = 0.186, *d* = 0.06
Male	56.3	33.3	37.0	40.8	65.1	39.0	2.71 (1.21)
Female	60.5	33.3	38.1	33.8	63.8	34.2	2.64 (1.26)
Age group, *overall statistical test*	χ^2^(3) = 4.36, *p* = 0.225, *V* = 0.05	χ^2^(3) = 14.19, *p* = 0.003, *V* = 0.09	χ^2^(3) = 52.84, *p* < 0.001, *V* = **0.17**	χ^2^(3) = 9.38, *p* = 0.025, *V* = 0.07	χ^2^(3) = 21.83, *p* < 0.001, *V* = **0.11**	χ^2^(3) = 40.15, *p* < 0.001, *V* = **0.15**	F(3, 1790) = 19.31, *p* < 0.001, *d* = **0.36**
50–59	59.9	32.4	45.6	36.3	68.2	43.1	2.86 (1.18)
60–69	55.5	39.4	41.6	37.3	68.1	40.2	2.82 (1.24)
70–79	58.3	31.0	27.8	31.6	59.6	29.9	2.38 (1.28)
≥ 80	62.1	27.7	25.6	43.0	55.4	24.6	2.38 (1.21)
School education, overall statistical test	χ^2^(3) = 8.47, *p* = 0.037, *V* = 0.07	χ^2^(3) = 12.85, *p* = 0.005, *V* = 0.09	χ^2^(3) = 47.18, *p* < 0.001, *V* = **0.16**	χ^2^(3) = 12.41, *p* = 0.006, *V* = 0.08	χ^2^(3) = 22.26, *p* < 0.001, *V* = **0.11**	χ^2^(3) = 10.09, *p* = 0.018, *V* = 0.08	F(3, 1763) = 20.36, *p* < 0.001, *d* = **0.37**
Low	59.2	26.3	30.8	33.5	54.8	33.5	2.38 (1.29)
Middle	61.3	31.9	28.6	32.3	61.7	32.7	2.48 (1.20)
Advanced	62.2	37.7	46.3	41.1	69.7	38.5	2.95 (1.19)
High	54.0	36.4	44.5	40.6	68.1	41.0	2.85 (1.23)
Net monthly household income, overall statistical test	χ^2^(4) = 10.49, *p* = 0.033, *V* = 0.08	χ^2^(4) = 12.49, *p* = 0.014, *V* = 0.09	χ^2^(4) = 34.00, *p* < 0.001, *V* = **0.15**	χ^2^(4) = 18.59, *p* < 0.001, *V* = **0.11**	χ^2^(4) = 9.35, *p* = 0.053, *V* = 0.08	χ^2^(4) = 9.21, *p* = 0.056, *V* = 0.08	F(4, 1597) = 15.17, *p* < 0.001, *d* = **0.39**
Low	50.1	26.6	29.1	31.3	54.5	31.9	2.23 (1.37)
Lower-middle	60.3	30.4	29.0	30.6	64.2	32.0	2.47 (1.16)
Middle	63.5	33.2	38.1	35.8	66.3	40.1	2.77 (1.25)
Upper-middle	62.0	34.6	42.2	43.4	64.6	38.8	2.86 (1.22)
High	58.8	39.0	45.9	41.8	66.0	39.6	2.91 (1.21)

Effect sizes in bold indicate small group differences

**Table 3:** Knowledge of diagnostic items of oral cancer by socio-demographic characteristics

Sample characteristics	The most common sites		Asympto-matic at early stages	Majority diagnosis at age 60+	Diagnose at an advanced stage	Appearance of early lesions	Sum score diagnostics
	Tongue	Floor of the mouth					
	%	%	%	%	%	%	*M* (*SD*)
Total	58.5	33.3	37.6	37.1	64.4	36.4	2.67 (1.24)
Tobacco consumption, overall statistical test	χ^2^(2) = 11.87, *p* = 0.003, *V* = 0.08	χ^2^(2) = 15.81, *p* < 0.001, *V* = 0.09	χ^2^(2) = 0.04, *p* = 0.978, *V* = 0.00	χ^2^(2) = 5.12, *p* = 0.077, *V* = 0.05	χ^2^(2) = 2.86, *p* = 0.239, *V* = 0.04	χ^2^(2) = 3.47, *p* = 0.177, *V* = 0.04	Welch F(2, 938.34) = 0.59, *p* = 0.553, *d* = 0.06
Smoker	67.0	25.0	36.8	32.2	68.1	32.5	2.62 (1.17)
Ex-smoker	57.7	32.8	37.6	37.0	63.5	38.3	2.67 (1.19)
Non-smoker/ occasional	56.2	37.1	37.3	39.3	63.1	37.3	2.70 (1.32)
Alcohol consumption, *overall statistical test*	χ^2^(6) = 4.58, *p* = 0.599, *V* = 0.05	χ^2^(6) = 5.10, *p* = 0.531, *V* = 0.05	χ^2^(6) = 8.53, *p* = 0.202, *V* = 0.07	χ^2^(6) = 12.28, *p* = 0.056, *V* = 0.08	χ^2^(6) = 7.21, *p* = 0.302, *V* = 0.06	χ^2^(6) = 7.67, *p* = 0.263, *V* = 0.07	Welch F(6, 663.18) = 1.48, *p* = 0.182, *d* = 0.14
Daily	50.9	37.2	28.3	41.7	65.0	28.8	2.52 (1.29)
Several times a week	58.2	34.5	38.6	42.8	68.1	36.5	2.79 (1.16)
About once a week	60.9	34.6	37.0	39.5	62.7	35.2	2.70 (1.21)
Several times a month	59.2	36.3	41.9	36.0	65.2	39.5	2.78 (1.25)
Once a month	60.9	30.8	39.8	31.5	68.8	32.7	2.64 (1.16)
Less often	58.1	29.6	37.5	32.7	60.4	39.6	2.58 (1.33)
Never	56.0	33.7	33.2	38.8	62.9	37.9	2.63 (1.27)

Effect sizes in bold indicate small group differences

### Knowledge of risk factors

Tables 4 and 5 show the detailed results for knowledge of oral cancer risk factors. On average, the participants correctly identified 4.05 (SD = 1.38) out of the seven risk factors. The risk factors tobacco consumption (92.0%) and prior oral cancer (87.7%) were widely recognised. Furthermore, 68.5% of the participants identified older age as a risk factor, and 59.0% identified alcohol. Only a minority knew that human papillomavirus (HPV) infection (36.4%), sun exposure (32.0%) and the low consumption of fruit and vegetables (29.9%) posed a risk for oral cancer

For the socio-demographic characteristics, small differences in the overall knowledge of risk factors were observed for age group (*d* = 0.28), school education (*d* = 0.36) and net monthly household income (*d* = 0.26). A post hoc test with Games–Howell correction showed that participants aged up to 69 years had a greater knowledge of risk factors than older participants (50s vs. 70s: *p* < 0.001, *d* = 0.31; 50s vs. ≥ 80: *p* < 0.001, *d* = 0.31; 60s vs. 70s: *p* = 0.001, *d* = 0.27; 60s vs. ≥ 80: *p* = 0.002, *d* = 0.27). A higher school education was associated with a higher level of knowledge of risk factors. Participants holding a low education level gave fewer correct answers than participants with a middle (Games–Howell correction; *p* = 0.002, *d * =−0.26), advanced (*p* < 0.001, *d * =−0.43) or high education level (*p* < 0.001, *d * =−0.51). The knowledge level of participants with a middle education level was lower than that of participants with a high education level (*p* < 0.001, *d *=− 0.23). For net monthly household income, some group differences were observed for the level of knowledge of risk factors. Participants living in households with a high net income had a higher knowledge level compared to those with a low to middle net income (Games–Howell correction; low: *p* < 0.001, *d * =−0.38; lower-middle: *p* = 0.016, *d * =−0.22; middle: *p* = 0.002, *d * =−0.27). In addition, the knowledge level of participants with an upper-middle net monthly household income was higher than that of those with a low income (*p* = 0.020, *d *=− 0.30)

For the awareness of the risk posed by the individual’s consumption habits, the knowledge of tobacco consumption was similarly high across the smoker, ex-smoker and non-smoker/occasional smoker categories (*p* = 0.946, *V* = 0.01). In the case of alcohol consumption, the awareness was generally moderate, with a slight difference across the consumption groups (*p* = 0.012, *V* = 0.10): only 45.1% of the participants who drank alcohol daily identified alcohol as a risk factor compared to 61.9% who drank alcohol once a week (*p* = 0.033, *V* = 0.15), or 65.7% never consuming alcohol (*p* = 0.006, *V* = 0.20)

SI File 2, Tables S4 and S5, provide results by region and post hoc tests of items with a relevant group difference based on the magnitude of the effect size

**Table 4:** Knowledge of oral cancer risk factors by socio-demographic characteristics

Sample characteristics	Older age	Alcohol consum-ption	Tobacco consump-tion		Low intake of fruit/veg-etables	Prior oral cancer lesion	HPV	Sun exposure	Sum score risk factors
	%	%	%		%	%	%	%	M (SD)
Total	68.5	59.0	92.0		29.9	87.7	36.4	32.0	4.05 (1.38)
Sex, *overall statistical test*	χ^2^(1) = 28.46, *p* < 0.001, *V* = **0.13**	χ^2^(1) = 0.91, *p* = 0.339, *V* = 0.02	χ^2^(1) = 7.30, *p* = 0.007, *V* = 0.06		χ^2^(1) = 0.00, *p* = 0.965, *V* = 0.00	χ^2^(1) = 0.07, *p* = 0.791, *V* = 0.01	χ^2^(1) = 4.52, *p* = 0.033, *V* = 0.05	χ^2^(1) = 3.35, *p* = 0.067, *V* = 0.04	t(1799) = 1.79, *p* = 0.074, *d* = 0.08
Male	74.7	57.8	93.8		29.8	87.5	33.8	34.1	4.11 (1.31)
Female	63.0	60.1	90.4		29.9	87.8	38.6	30.1	4.00 (1.43)
Age group, *overall statistical test*	χ^2^(3) = 28.69, *p* < 0.001, *V* = **0.13**	χ^2^(3) = 8.94, *p* = 0.030, *V* = 0.07	χ^2^(3) = 9.79, *p* = 0.020, *V* = 0.07		χ^2^(3) = 2.09, *p* = 0.553, *V* = 0.03	χ^2^(3) = 45.89, *p* < 0.001, *V* = **0.16**	χ^2^(3) = 12.34, *p* = 0.006, *V* = 0.08	χ^2^(3) = 11.26, *p* = 0.010, *V* = 0.08	Welch F(3, 842.18) = 11.06, *p* < 0.001, *d* = **0.28**
50–59	75.0	62.2	93.7		31.2	92.6	40.0	28.0	4.23 (1.33)
60–69	69.8	61.2	93.1		30.9	90.7	37.9	32.8	4.16 (1.31)
70–79	60.6	54.5	90.5		28.0	81.7	34.9	31.0	3.81 (1.38)
≥ 80	61.5	54.6	88.5		27.7	80.3	28.7	38.6	3.80 (1.50)
School education, *overall statistics test*	χ^2^(3) = 51.51, *p* < 0.001, *V* = **0.17**	χ^2^(3) = 13.48, *p* = 0.004, *V* = 0.09	χ^2^(3) = 18.36, *p* < 0.001, *V* = **0.10**		χ^2^(3) = 0.23, *p* = 0.973, *V* = 0.01	χ^2^(3) = 39.66, *p* < 0.001, *V* = **0.15**	χ^2^(3) = 20.36, *p* < 0.001, *V* = **0.11**	χ^2^(3) = 5.19, *p* = 0.158, *V* = 0.05	Welch F(3, 877.63) = 18.19, *p* < 0.001, *d* = **0.36**
Low	58.9	51.4	87.7		29.1	78.2	27.0	29.8	3.62 (1.45)
Middle	61.4	59.5	90.5		30.8	87.8	35.9	32.7	3.99 (1.40)
Advanced	73.2	65.4	94.1		30.4	91.4	39.1	27.8	4.21 (1.31)
High	77.6	59.6	94.8		29.9	91.6	41.9	34.4	4.30 (1.27)
Net monthly household income, *overall statistical test*	χ^2^(4) = 22.30, *p* < 0.001, *V* = **0.12**	χ^2^(4) = 11.45, *p* = 0.022, *V* = 0.08	χ^2^(4) = 24.12, *p* < 0.001, *V* = **0.12**		χ^2^(4) = 3.60, *p* = 0.463, *V* = 0.05	χ^2^(4) = 24.95, *p* < 0.001, *V* = **0.12**	χ^2^(4) = 6.24, *p* = 0.182, *V* = 0.06	χ^2^(4) = 7.19, *p* = 0.126, *V* = 0.07	Welch F(4, 691.18) = 6.67, *p* < 0.001, *d* = **0.26**
Low	63.7	50.8	86.3		28.3	79.2	30.5	38.6	3.77 (1.61)
Lower-middle	62.3	61.3	90.0		32.5	87.3	38.0	29.2	4.01 (1.40)
Middle	67.5	55.1	92.5		26.1	88.4	34.0	30.9	3.94 (1.35)
Upper-middle	70.0	62.1	93.0		28.9	93.3	40.2	32.5	4.20 (1.25)
High	76.4	62.2	96.6		30.4	90.6	37.9	35.2	4.29 (1.22)

Effect sizes in bold indicate small group differences. HPV is the abbreviation for human papillomavirus

**Table 5:** Knowledge of oral cancer risk factors by socio-demographic characteristics

Sample characteristics	Older age	Alcohol consum-ption	Tobacco consump-tion	Low intake of fruit/veg-etables		Prior oral cancer lesion	HPV	Sun exposure	Sum score risk factors
	%	%	%		%	%	%	%	M (SD)
Total	68.5	59.0	92.0		29.9	87.7	36.4	32.0	4.05 (1.38)
Tobacco consumption, *overall statistical test*	χ^2^(2) = 1.79, *p* = 0.408, *V* = 0.03	χ^2^(2) = 4.15, *p* = 0.125, *V* = 0.05	χ^2^(2) = 0.11, *p* = 0.946, *V* = 0.01		χ^2^(2) = 15.28, *p* < 0.001, *V* = 0.09	χ^2^(2) = 1.73, *p* = 0.421, *V* = 0.03	χ^2^(2) = 1.34, *p* = 0.511, *V* = 0.03	χ^2^(2) = 11.42, *p* = 0.003, *V* = 0.08	F(2, 1777) = 0.48, *p* = 0.617, *d* = 0.06
Smoker	69.5	55.2	91.9		37.8	86.3	34.0	24.5	3.99 (1.39)
Ex-smoker	66.6	61.6	91.6		29.8	87.7	37.6	33.2	4.08 (1.39)
Non-smoker/occasional	69.6	58.1	92.1		26.1	89.0	36.9	34.6	4.06 (1.37)
Alcohol consumption, *overall statistical test*	χ^2^(6) = 16.39, *p* = 0.012, *V* = 0.10	χ^2^(6) = 16.30, *p* = 0.012, *V* = 0.10	χ^2^(6) = 9.78, *p* = 0.134, *V* = 0.07		χ^2^(6) = 9.35, *p* = 0.155, *V* = 0.07	χ^2^(6) = 31.46, *p* < 0.001, *V* = **0.13**	χ^2^(6) = 11.14, *p* = 0.084, *V* = 0.08	χ^2^(6) = 10.43, *p* = 0.108, *V* = 0.08	Welch F(6, 664.44) = 2.49, *p* = 0.022, *d* = 0.19
Daily	67.9	45.1	93.2		25.5	79.2	32.6	37.3	3.81 (1.39)
Several times a week	74.7	59.8	91.9		30.8	89.9	31.8	34.7	4.14 (1.28)
About once a week	69.6	61.9	92.6		28.1	91.2	39.8	31.1	4.14 (1.33)
Several times a month	65.4	57.5	93.0		29.0	94.5	33.2	36.1	4.09 (1.30)
Once a month	66.5	54.8	92.5		29.3	85.0	40.1	28.6	3.97 (1.34)
Less often	62.5	59.0	88.4		27.4	84.9	40.4	27.1	3.90 (1.51)
Never	73.7	65.7	95.0		37.9	83.1	33.2	34.6	4.23 (1.40)

Effect sizes in bold indicate small group differences. HPV is the abbreviation for human papillomavirus

## Discussion

Our results show that the public awareness of oral cancer among older German adults remains low. To conceptualise a national awareness campaign, we explored the public’s knowledge of the diagnostic signs, symptoms and risk factors of oral cancer, as preventive measures may not be effective without a sufficient knowledge base. Overall, this study shows that the public’s level of knowledge of diagnostic signs, symptoms and risk factors is unsatisfactory. To contextualise our findings within the current literature, we considered previous studies that employed thematically similar questionnaires. It is important to note that our sample consisted of individuals aged 50 to over 80 – the primary age group affected by oral cancer in Germany. In contrast, all studies cited in the discussion also included participants aged 18 to 49

The overall level of public knowledge of diagnostic aspects was low, consistent with findings from the UK (Niksic et al. 2015; Al-Kaabi et al. 2016). For example, when asked about the clinical signs of an early lesion, only about one-third of the German respondents knew the correct answer. Comparable results (approximately 33%) were reported in other studies (Agrawal et al. 2012; Hertrampf et al. 2012; Suárez-Fernández et al. 2023), and some studies found even lower rates, ranging from 13 to 27% (Al-Maweri et al. 2014; Al-Kaabi et al. 2016; Nocini et al. 2020). For the two main sites, only 59% correctly identified the tongue as a common localisation and only one-third the floor of the mouth; even lower figures were reported in a study from northern Germany (tongue 43% vs. floor of mouth 20%) (Hertrampf et al. 2012)

The overall knowledge of risk factors was medium, with considerable differences in the awareness of specific factors. Interestingly, all of the studies cited below report significantly higher public awareness of tobacco compared to alcohol consumption. In this study, 92% of respondents identified tobacco consumption as a risk factor, whereas only 59% did so for alcohol. International studies report awareness rates for alcohol of between 80 and 90%, compared to 40 to 59% for alcohol (Dost et al. 2016; Kawecki et al. 2019; Nocini et al. 2020; Grossmann et al. 2021; Suárez-Fernández et al. 2023; Merdad et al. 2025). When tobacco-related knowledge dropped to 72 to 76%, alcohol-related knowledge also declined (34 to 59%) (Agrawal et al. 2012; Hertrampf et al. 2012; Al-Maweri et al. 2014; Azimi et al. 2019). In studies with particularly low tobacco awareness (29 to 55%), knowledge of alcohol as a risk factor waseven lower (15 to 31%) (Monteiro et al. 2016; Amarasinghe et al. 2010; Kassim et al. 2017; Zhou et al. 2022). The exception to this pattern is the study by Wimardhani et al. (2019), in which all respondents correctly identified tobacco and alcohol consumption as risk factors. The higher awareness of tobacco consumption as a risk factor was likely attributable to the longstanding and widespread health campaigns and media coverage of its negative health effects (e.g. graphic health warnings on tobacco packaging) (Drovandi et al. 2019). Moreover, some of the studies surveyed populations in which alcohol was restricted or uncommon due to religious or cultural norms, potentially lowering the relevance of alcohol as a risk factor. However, given the synergistic effect of tobacco and alcohol in increasing the risk of developing oral cancer, public education should urgently address both risk factors

Regarding the risk factor ‘older age’, this study and international studies have revealed a clear lack of knowledge. In addition, this study noted the tendency of older participants (70 years and older compared to the age group of 50 to 59 years) being less aware of older age as a risk factor, making this a relevant target group for an awareness campaign. However, when interpreting the results, it should be borne in mind that term ‘older age’ is inherently ambiguous. Whereas in this study, 69% of the respondents acknowledged older age as a risk factor, only 37% named ≥ 60 years as the primary age group for oral cancer diagnoses in Germany. This distinction is relevant, as age-related risk varies by country. For example, an Iranian study reported that only 18% of respondents were aware of ≥ 50 years being the main risk group (Azimi et al. 2019), whereas in a Chinese study, the majority recognised 40 to 60 years as the main at-risk age group (Zhou et al. 2022). Studies that asked about older age without age specification showed awareness levels of between 26 and 55% (Hertrampf et al. 2012; Al-Maweri et al. 2014; Kassim et al. 2017; Merdad et al. 2025)

As the findings of this study will inform the development of a national awareness campaign, identifying relevant target groups in the population is essential. In this study, respondents with an older age, a lower education level or income achieved lower knowledge scores than respondents with a higher education level or income. These results are consistent with both older and more recent international studies on age and school education (Patton et al. 2004; Tomar and Logan 2005; Hertrampf et al. 2012; Kassim et al. 2017; Nocini et al. 2020; Varela-Centelles et al. 2021; Merdad et al. 2025) as well as income (Anderson et al. 2008; Dalton et al. 2008; Dost et al. 2016; Merdad et al. 2025)

For the risk habits, tobacco and alcohol consumption were more prevalent among the male respondents of this study. Current smoking was more common among younger participants (50–69 years) and those with a low education, whereas consuming alcohol with a high frequency was more common among older respondents. Regardless of gender and the individual smoking behaviour, an awareness of tobacco as a risk factor was high, whereas an awareness of alcohol was considerably lower, calling for alcohol as a risk factor to be addressed more intensively. This pattern of regular smokers’ risk awareness is consistent with similar studies (Monteiro et al. 2016; Kawecki et al. 2019; Wimardhani et al. 2019). In contrast to the pattern for tobacco consumption, the risk awareness of alcohol consumption was generally low, and especially so for daily alcohol drinkers in this study. A low risk awareness in heavy alcohol consumers was also found in other studies (Amarasinghe et al. 2010; Monteiro et al. 2016; Wimardhani et al. 2019; Varela-Centelles et al. 2021), but in one study, alcohol was a well-known risk factor among daily alcohol consumers (Kawecki et al. 2019)

In summary, the findings indicate that, beyond objective risk factors such as age, tobacco and alcohol consumption, the socioeconomic variables of education level and income should be considered as key target dimensions in tailoring a national awareness campaign focused on diagnostic knowledge and the risk factors of oral cancer. One focus of the national awareness campaign should be addressing the at-risk groups of older people and those with a critical alcohol consumption habit – as both groups lack an awareness of their respective risk-increasing factors of oral cancer

## Limitations

As with all CATI-based surveys, distortions may arise due to non-coverage in the target population without landline access and the increasing reliance on mobile phones. To minimise such bias, the opinion research institute forsa. used the recognised OmniTel^®^ system based on the ‘ADM telephone sample’ described in the methods section. In addition, potential self-selection bias must be considered, as individuals with a greater interest in the topic were likely to be more willing to participate. Also, self-reported data can be subject to social desirability bias and participants’ stated knowledge may not fully reflect their actual knowledge or behaviour. The survey excluded non-German speakers, which limits generalisability to the entire population of Germany aged 50 and older

## Conclusion

This national survey demonstrates that the German population is not sufficiently informed about signs, symptoms and risk factors of oral cancer. Knowledge deficits were in particular associated with older age, lower education levels and low income. These findings provide a foundation to design, implement and evaluate a national awareness campaign aimed at informing the public, especially vulnerable subgroups, about these types of tumours, the diagnostic signs and specific risk factors, and preventive measures

## Supplementary Information

Below is the link to the electronic supplementary material.

Supplementary Material 1

Supplementary Material 2

## Data Availability

The datasets generated during and/or analysed during the current study are available from the corresponding author on reasonable request.
