# Supplementary material for: Self-reported awareness of the prevention and early detection of oral cancer: a survey of 50+ year old people in Germany
Source: J Cancer Res Clin Oncol. 2026 May 18;152(5):110. doi: 10.1007/s00432-026-06491-z (PMC13187086; doi:10.1007/s00432-026-06491-z)
Supplement: Supplementary file 1 — Supplementary Material 1 [file 432_2026_6491_MOESM1_ESM.docx]

**Supplement Information – File 1**

# National public survey on the topic of oral cancer (English version)

We are currently conducting a representative survey in Germany on the topic of health as part of a scientific project. We would be grateful if you could answer a few questions on this important topic.

## **I. Level of knowledge and awareness**

1. [perception of issue; O01] **Have you heard or read anything about oral cancer in the last six months?**

- Yes
- No

DO NOT READ ALOUD

- I don’t know
- No information provided

1. [subjective level of knowledge] **And how well informed are you currently about oral cancer?**

**Please rate your level of knowledge on a scale of 0 to 100.**

**0 means that you know nothing about oral cancer. 100 means that you know everything there is to know about oral cancer. At 100, you have a similar level of knowledge to a professional.**

INT: INTEGER VALUE BETWEEN 0 AND 100

______

DO NOT READ ALOUD

- I don’t know
- No information provided

1. [subjective level of knowledge; NEU_DLK] **Think again about the scale from 0 to 100 you just used. How much knowledge do you think you need to be able to deal appropriately with the risks of oral cancer or oral cancer disease?**

INT: INTEGER VALUE BETWEEN 0 AND 100

______

DO NOT READ ALOUD

- I don’t know
- No information provided

1. [knowledge of diagnostic items; O02] **What do you think: excluding the lips, which of the following are the two most common sites of oral cancer?**

INT: PLEASE READ OUT IN ITS ENTIRETY; TWO ANSWER OPTIONS POSSIBLE; RANDOMISE

- Soft palate, i.e. the area where the soft palate and uvula are located
- Tongue
- Gingiva
- Buccal mucosa
- Floor of mouth, i.e. the area under the tongue

DO NOT READ ALOUD

- I don’t know
- No information provided

1. [knowledge of diagnostic items; O03] **What do you think: What is the most common sign that patients notice in the early stages of the disease?**

**The early stages refer to the period at the onset of the disease.**

INT: PLEASE READ OUT LOUD IN ITS ENTIRETY; ONE ANSWER OPTION POSSIBLE; RANDOMISE 1 TO 3

- Pain
- Ulceration
- Swelling
- None of the above; patients do not notice that they have oral cancer in the early stages

DO NOT READ ALOUD

- I don’t know
- No information provided

1. [knowledge; O04] **What do you think: In which of the following age groups are most cases of oral cancer diagnosed by doctors?**

INT: PLEASE READ OUT IN ITS ENTIRETY; ONE ANSWER OPTION POSSIBLE

- Less than 18 years of age
- 18–39 years of age
- 40–59 years of age
- 60 years of age or older

DO NOT READ ALOUD

- I don’t know
- No information provided

1. [knowledge of diagnostic items; O06] **What do you think: At what stage is oral cancer most commonly diagnosed by doctors?**

INT: PLEASE READ OUT IN ITS ENTIRETY; ONE ANSWER OPTION POSSIBLE

- In the premalignat stage, i.e. when there are signs that oral cancer could develop
- In the early stage, i.e. at the onset of the disease
- In the advanced stage

DO NOT READ ALOUD

- I don’t know
- No information provided

1. [knowledge of risk factors; O07] **I will now read you a few statements, only one of which is correct. Which one do you think is the correct statement?**

**Cancer of the lips …**

INT: PLEASE READ OUT IN ITS ENTIRETY; ONE ANSWER OPTION POSSIBLE; RANDOMISE

- Are related to how long the lips are exposed to the sun
- Are increasing every year
- Have a worse prognosis than most oral cancers
- Affect the upper lip more frequently than the lower lip
- Have not been related to any form of tobacco use

DO NOT READ ALOUD

- I don’t know
- No information provided

1. [knowledge of diagnostic items; O08] **What do you think: How does oral cancer usually appear in its early stages, i.e. at the onset of the disease?**

INT: PLEASE READ OUT IN ITS ENTIRETY; ONE ANSWER OPTION POSSIBLE; RANDOMISE

- As a small painless red area
- As a small painful red area
- As a small painful white area
- As a small bleeding area

DO NOT READ ALOUD

- I don’t know
- No information provided

1. [knowledge of risk factors; O10] **What do you think: Which of the following factors increase the risk of developing oral cancer?**

PLEASE ENQUIRE ABOUT THE FOLLOWING CATEGORIES; RANDOMISE

1. Older age

2. Use of alcohol

3. Use of tobacco

4. Family history of cancer

5. Low consumption of fruit and vegetables

6. Prior oral cancer lesion

7. Poor-fitting dentures

8. Poor oral hygiene

9. Consumption of spicy foods

10. Human papillomavirus

11. Hot beverages and foods

12. Obesity, i.e. morbidly overweight

- Yes
- No
- I don’t know
- No information provided

## **II Role of the dentist**

In the following section, we will ask you questions about visiting a dentist.

1. [visit to the dentist; O15] **When was the last time you saw a dentist?**

INT: PLEASE READ OUT IN ITS ENTIRETY; ONE ANSWER OPTION POSSIBLE

- Within the last three months
- Within the last six months
- Within the last year
- Or was it more than a year ago

DO NOT READ ALOUD

- I never go to the dentist
- I don’t remember
- No information provided

1. [visit to the dentist; O16] *[Filter: If the respondent did not answer “I never go to the dentist” to question 11/O15]*

**What was the reason for your last visit to the dentist?**

INT: PLEASE READ ALOUD; MULTIPLE ANSWER OPTIONS POSSIBLE; RANDOMISE 1 TO 4

- Annual/Six-monthly check-up
- I am currently undergoing treatment
- I had acute symptoms
- Professional teeth cleaning

DO NOT READ ALOUD

- Other reason
- I don’t remember
- No information provided

1. [content medical history; O9] *[Filter: If the respondent did not answer “I never go to the dentist” to question 11/O15]*

**Which of the following information does your dentist collect when taking your medical history?**

PLEASE ASK ABOUT THE FOLLOWING CATEGORIES; RANDOMISE

1. Past alcohol use
2. Present alcohol use
3. Type and amount of alcohol used
4. Previous tobacco use
5. Present tobacco use
6. Type and amount of tobacco
7. Your history of cancer
8. Family history of cancer

- Yes
- No
- I don’t know
- No information provided

1. [content check-up; O17] *[Filter: If the respondent did not answer “I never go to the dentist” to question 11/O15]*

**What do you think: What does your dentist usually look at during your check-up?**

INT: PLEASE READ ALOUD; MULTIPLE ANSWERS POSSIBLE; RANDOMISE

- The teeth
- The oral mucosa
- Dental prostheses (e.g. dentures)
- The gingiva
- The lymph nodes
- The tongue

DO NOT READ ALOUD

- Other
- I don’t go for regular check-ups
- I don’t know
- No information provided

1. [communication with dentist; O18] *[Filter: If the respondent did not answer “I never go to the dentist” to question 11/O15]*

**How precisely does your dentist usually explain what he or she is looking at during the examination?**

**She or he …**

INT: PLEASE READ OUT IN ITS ENTIRETY; ONE ANSWER OPTION POSSIBLE

- Usually says nothing at all
- Gives a rough description
- Explains it in great detail

DO NOT READ ALOUD

- I don’t know
- No information provided

1. [communication with dentist; O19] *[Filter: If the respondent did not answer “I never go to the dentist” to question 11/O15]*

**How exactly do you ask your dentist what he or she is looking at during the examination?**

INT: PLEASE READ OUT IN ITS ENTIRETY; ONE ANSWER OPTION POSSIBLE

- I usually don’t ask my dentist any questions.
- I want to have a general idea and ask about it.
- I want to know exactly what is going on and ask a lot of questions.

DO NOT READ ALOUD

- I don’t know
- No information provided

Now, regardless of your visit to the dentist, we would like to take a closer look at the examination of your oral mucosa.

1. [oral mucosa examination; O20] **Have your oral mucosa ever been medically examined?**

- Yes
- No

DO NOT READ ALOUD

- I don’t know
- No information provided

1. [oral mucosa examination; O21] *[Filter: If the respondent answered “Yes” to question 17/O20]* **Who last examined your oral mucosa?**

INT: PLEASE READ OUT IN ITS ENTIRETY; ONE ANSWER OPTION POSSIBLE; RANDOMISE 1 TO 4

- By a dentist
- By a general practitioner
- By an ear, nose and throat specialist
- By a dermatologist
- By another doctor

DO NOT READ ALOUD

- I don’t know
- No information provided

1. [oral mucosa examination; O22] **If you had pain or problems with the mucous membranes in your mouth, who would you turn to first?**

INT: PLEASE READ OUT IN ITS ENTIRETY; ONE ANSWER OPTION POSSIBLE; RANDOMISE 1 TO 4

- A dentist
- A general practitioner
- An ear, nose and throat specialist
- A dermatologist
- Another doctor

DO NOT READ ALOUD

- I don’t know
- No information provided

## **III Risk perception and information behaviour**

In the following, we would like to learn about your assessment of oral cancer and how you would obtain information on this topic.

1. [risk perception] **How likely do you think it is that you will develop oral cancer at some point in the future?**

**Please rate your probability on a scale from 0 to 100.**

**0 means that you consider it extremely unlikely that you will develop oral cancer. 100 means that you consider it extremely likely that you will develop oral cancer.**

INT: INTEGER VALUE BETWEEN 0 AND 100

______

DO NOT READ ALOUD

- I don’t know
- No information provided

1. [risk perception] **If you were to develop oral cancer at some point in the future, how severe would the disease be for you?**

**Please rate the severity of the disease on a scale of 0 to 100.**

**0 means that oral cancer would be completely harmless. 100 means that the disease would be extremely severe.**

INT: INTEGER VALUE BETWEEN 0 AND 100

______

DO NOT READ ALOUD

- I don’t know
- No information provided

1. [information behaviour] **Now think about how you deal with information on the subject of oral cancer.**

**I will read you a few statements on this topic. Please answer with “strongly agree”, “somewhat agree”, “neither agree nor disagree”, “somewhat disagree” or “strongly disagree”.**

PLEASE ASK FOR THE FOLLOWING ITEMS; RANDOMISE

1. I avoid learning everything about oral cancer.
2. Even if it upsets me, I want to know everything about oral cancer.
3. I want to know everything about oral cancer.
4. When it comes to oral cancer, sometimes ignorance is bliss.
5. I can think of situations in which I would rather not know everything about oral cancer.
6. It is important to know everything about oral cancer.
7. Strongly agree
8. Somewhat agree
9. Neither agree nor disagree
10. Somewhat disagree
11. Strongly disagree

DO NOT READ ALOUD

1. I don’t know
2. No information provided
3. [information behaviour; O25] **If you had cancer yourself, which of these groups of people would be particularly important for you to talk to?**

INT: PLEASE READ OUT; MULTIPLE ANSWERS POSSIBLE; RANDOMISE

- My dentist
- My general practitioner
- My ear, nose and throat specialist
- My dermatologist
- My partner
- My closest friends
- My closest relatives
- Other people affected

DO NOT READ ALOUD

- Other
- No one; I wouldn’t want to talk to anyone at first
- I don’t know
- No information provided

1. [information behaviour; O27] **Do you think that the media should report more frequently on the topic of oral cancer? By media, we mean television, radio, newspapers, magazines and the internet.**

- Yes
- No

DO NOT READ ALOUD

- I don’t know/I don’t care
- No information provided

## **IV Personal information**

Almost done! Now we would like to learn more about you.

1. [cancer involvement; O12] **Are you or have you ever been affected by cancer?**

- Yes
- No

DO NOT READ ALOUD

- I don’t know
- No information provided

1. [cancer involvement; O13] **Are there or have there been one or several cases of cancer in your immediate family or circle of friends?**

- Yes, several
- Yes, one
- No

DO NOT READ ALOUD

- I don’t know
- No information provided

1. [health behaviour; O14] **We have collected some statements about what other people say about their own health and health behaviour.**

**To what extent do these statements apply to you personally? Please answer with “****strongly agree”, “somewhat agree”, “neither agree nor disagree”, “somewhat disagree” or “strongly disagree”.**

PLEASE ASK FOR THE FOLLOWING ITEMS; RANDOMISE

1. I am a health-conscious person.
2. I always take advantage of cancer-screening opportunities.
3. By living a healthy lifestyle, I can significantly reduce my risk of developing cancer.
4. I have no influence over whether or not I develop cancer.
5. I only go to the doctor when I have severe symptoms.
6. There are some things I simply don’t want to give up, even if they are bad for my health.
7. To reduce my risk of cancer, I would be willing to live a healthier lifestyle, for example, by drinking less alcohol, smoking less tobacco and eating more fruit and vegetables.

- Strongly agree
- Somewhat agree
- Neither agree nor disagree
- Somewhat disagree
- Strongly disagree

DO NOT READ ALOUD

- I don’t know
- No information provided

1. [tobacco consumption; O28] **How would you describe yourself at present: As …**

INT: PLEASE READ OUT LOUD

- A regular smoker
- An occasional smoker
- A non-smoker

DO NOT READ ALOUD

- I don’t know
- No information provided

1. [tobacco consumption; O29] *[Filter: If “non-smoker” was answered in question O28]* **Did you used to smoke, at least occasionally?**

- Yes
- No

DO NOT READ ALOUD

- No information provided

1. [tobacco consumption; O30] *[Filter: If “regular smoker” or “occasional smoker” was answered in question O28]* **When did you start smoking, at least occasionally? Please tell me how old you were at that time.**

INT: PLEASE ASSIGN STATEMENT

- Up to 10 years old
- 10 to 19 years old
- 20 to 29 years old
- 30 to 39 years old
- 40 to 49 years old
- 50 to 59 years old
- 60 years old or older

DO NOT READ ALOUD

- I don’t know
- No information provided

1. [tobacco consumption; O31] *[Filter: If the answer to question O28 was “regular smoker” or “occasional smoker”]* **How many cigarettes do you currently smoke on average per day?**

INT: PLEASE READ OUT

- Less than 1
- 1 to 9
- 10 to 19
- 20 to 29
- 30 or more
- I do not smoke cigarettes

DO NOT READ ALOUD

- I don’t know
- No information provided

1. [alcohol consumption; O32] **If you think back over the last 12 months, how often did you drink alcohol? That includes beer, wine, sparkling wine, spirits, alcoholic mixed drinks or similar.**

INT: PLEASE READ OUT

- Daily
- Several times a week
- About once a week
- Several times a month
- Once a month
- Less often
- Never

DO NOT READ ALOUD

- I don’t know
- No information provided

## Sociodemographic information provided by forsa.

[ges] Sex

- Male
- Female

[alter] Age

Integer value ______

[schul] School education

- Without secondary school/primary school leaving certificate
- Secondary school/primary school leaving certificate
- Intermediate school leaving certificate, secondary school leaving certificate, technical college entrance qualification
- Polytechnic secondary school leaving certificate (8th/10th grade)
- Technical college entrance qualification, technical college diploma
- general or subject-specific university entrance qualification
- Technical college/university studies
- Other school qualification
- No information provided

[eink] Net monthly household income

- Less than 500 EUR
- 500 to less than 1,000 EUR
- 1,000 to less than 1,500 EUR
- 1,500 to less than 2,000 EUR
- 2,000 to less than 2,500 EUR
- 2,500 to less than 3,000 EUR
- 3,000 to less than 3,500 EUR
- 3,500 to less than 4,000 EUR
- 4,000 to less than 4,500 EUR
- 4,500 or above
- No information provided

[hhg] Household size

Integer value ______

[bula] Federal state

- Schleswig-Holstein
- Hamburg
- Lower Saxony
- Bremen
- North Rhine-Westphalia
- Hesse
- Rhineland-Palatinate
- Baden-Württemberg
- Bavaria
- Saarland
- Berlin
- Brandenburg
- Mecklenburg-Western Pomerania
- Saxony
- Saxony-Anhalt
- Thuringia

[einw] City size

Integer value ______
