# Supplementary material for: Self-reported awareness of the prevention and early detection of oral cancer: a survey of 50+ year old people in Germany
Source: J Cancer Res Clin Oncol. 2026 May 18;152(5):110. doi: 10.1007/s00432-026-06491-z (PMC13187086; doi:10.1007/s00432-026-06491-z)
Supplement: Supplementary file 2 — Supplementary Material 2 [file 432_2026_6491_MOESM2_ESM.docx]

# Supplement Information – File 2

[Table S1 Complete characteristics of study population by sociodemographic group 2](#_Toc221782214)

[Table S2 Knowledge of diagnostic items of oral cancer by region 7](#_Toc221782215)

[Table S3 Results for post hoc tests on knowledge of diagnostic items of oral cancer by sociodemographic group 8](#_Toc221782216)

[Table S4 Knowledge of oral cancer risk factors by region 11](#_Toc221782217)

[Table S5 Results for post hoc tests on knowledge of oral cancer risk factors by 12](#_Toc221782218)

[sociodemographic group 12](#_Toc221782219)

[References 14](#_Toc221782220)

### Table S1 Complete characteristics of study population by sociodemographic group

| **Characteristics** | **Total** | **Sex** | | **Age group** | | | | **School education** | | | | **Net monthly household income** | | | | |
| --- | --- | --- | --- | --- | --- | --- | --- | --- | --- | --- | --- | --- | --- | --- | --- | --- |
|  |  | Male | Fe-male | 50–59 | 60–69 | 70–79 | ≥ 80 | Low | Mid-dle | Adva-nced | High | Low | Lowermid-dle | Mid-dle | Uppermid-dle | High |
|  | n (%) | n (%) | n (%) | n (%) | n (%) | n (%) | n (%) | n (%) | n (%) | n (%) | n (%) | n (%) | n (%) | n (%) | n (%) | n (%) |
| **Sex**, *χ²*-test |  | n/a | | χ²(3) = 11.77, *p* = .008, *V* = 0.08 | | | | χ²(3) = 10.07, *p* = .018, *V* = 0.08 | | | | χ²(4) = 40.69, *p* < .001, *V* = **0.16** | | | | |
| Male | 845 (46.9) | 845 (100.0) | 0 (0.0) | 306 (50.0) | 262 (48.6) | 147 (45.6) | 124 (38.8) | 144 (45.3) | 228 (43.6) | 159 (43.7) | 293 (52.0) | 74 (36.8) | 156 (39.8) | 145 (44.2) | 116 (47.0) | 256 (58.6) |
| Female | 956 (53.1) | 0 (0.0) | 956 (100.0) | 306 (50.0) | 277 (51.4) | 176 (54.4) | 196 (61.2) | 174 (54.7) | 296 (56.4) | 204 (56.3) | 270 (48.0) | 126 (63.2) | 236 (60.2) | 183 (55.8) | 130 (53.0) | 181 (41.4) |
| **Age group**,  *χ²*-test |  | χ²(3) = 11.77, *p* = .008, *V* = 0.08 | | n/a | | | | χ²(9) = 77.23, *p* < .001, *V* = **0.12** | | | | χ²(12) = 204.01, *p* < .001, *V* = **0.21** | | | | |
| 50–59 | 612 (34.1) | 306 (36.5) | 306 (32.1) | 612 (100.0) | 0 (0.0) | 0 (0.0) | 0 (0.0) | 58 (18.4) | 170 (32.5) | 161 (44.4) | 219 (38.9) | 27 (13.5) | 87 (22.2) | 100 (30.5) | 95 (38.7) | 241 (55.1) |
| 60–69 | 539 (30.1) | 262 (31.2) | 277 (29.0) | 0 (0.0) | 539 (100.0) | 0 (0.0) | 0 (0.0) | 102 (32.1) | 165 (31.5) | 109 (29.9) | 160 (28.4) | 78 (38.8) | 102 (26.0) | 100 (30.4) | 83 (33.6) | 124 (28.3) |
| 70–79 | 323 (18.0) | 147 (17.5) | 176 (18.4) | 0 (0.0) | 0 (0.0) | 323 (100.0) | 0 (0.0) | 67 (21.3) | 96 (18.4) | 52 (14.2) | 105 (18.7) | 43 (21.8) | 94 (24.1) | 76 (23.1) | 40 (16.1) | 36 (8.2) |
| ≥ 80 | 320 (17.8) | 124 (14.8) | 196 (20.5) | 0 (0.0) | 0 (0.0) | 0 (0.0) | 320 (100.0) | 90 (28.3) | 92 (17.7) | 42 (11.5) | 79 (14.0) | 52 (25.9) | 108 (27.7) | 52 (16.0) | 28 (11.6) | 37 (8.4) |
| **School education**,  *χ²*-test |  | χ²(3) = 10.07, *p* = .018, *V* = 0.08 | | χ²(9) = 77.23, *p* < .001, *V* = **0.12** | | | | n/a | | | | χ²(12) = 248.82, *p* < .001, *V* = **0.23** | | | | |
| Low | 318 (18.0) | 144 (17.5) | 174 (18.4) | 58 (9.6) | 102 (19.0) | 67 (21.0) | 90 (29.6) | 318 (100.0) | 0 (0.0) | 0 (0.0) | 0 (0.0) | 72 (38.3) | 101 (26.3) | 55 (16.9) | 32 (13.2) | 18 (4.0) |
| Middle | 524 (29.6) | 228 (27.7) | 296 (31.3) | 170 (27.9) | 165 (30.8) | 96 (30.0) | 92 (30.6) | 0 (0.0) | 524 (100.0) | 0 (0.0) | 0 (0.0) | 67 (35.4) | 141 (36.7) | 112 (34.6) | 73 (29.8) | 80 (18.5) |
| Advanced | 363 (20.6) | 159 (19.3) | 204 (21.7) | 161 (26.5) | 109 (20.3) | 52 (16.1) | 42 (13.8) | 0 (0.0) | 0 (0.0) | 363 (100.0) | 0 (0.0) | 31 (16.6) | 60 (15.6) | 61 (18.9) | 44 (18.1) | 112 (25.8) |
| High | 563 (31.8) | 293 (35.5) | 270 (28.6) | 219 (36.0) | 160 (29.9) | 105 (32.8) | 79 (26.0) | 0 (0.0) | 0 (0.0) | 0 (0.0) | 563 (100.0) | 18 (9.7) | 83 (21.4) | 96 (29.6) | 95 (38.9) | 225 (51.7) |
| **Net monthly household income**, *χ²*-test |  | χ²(4) = 40.69,  *p* < .001,  *V* = **0.16** | | χ²(12) = 204.01,  *p* < .001, *V* = **0.21** | | | | χ²(12) = 248.82,  *p* < .001, *V* = **0.23** | | | | n/a | | | | |
| Low | 200 (12.5) | 74 (9.9) | 126 (14.7) | 27 (4.9) | 78 (16.0) | 43 (15.0) | 52 (18.6) | 72 (26.0) | 67 (14.1) | 31 (10.1) | 18 (3.5) | 200 (100.0) | 0 (0.0) | 0 (0.0) | 0 (0.0) | 0 (0.0) |
| Lower-middle | 392 (24.4) | 156 (20.9) | 236 (27.5) | 87 (15.8) | 102 (21.0) | 94 (32.6) | 108 (39.1) | 101 (36.4) | 141 (29.9) | 60 (19.4) | 83 (16.0) | 0 (0.0) | 392 (100.0) | 0 (0.0) | 0 (0.0) | 0 (0.0) |
| Middle | 328 (20.5) | 145 (19.4) | 183 (21.4) | 100 (18.2) | 100 (20.5) | 76 (26.2) | 52 (18.9) | 55 (19.7) | 112 (23.7) | 61 (19.8) | 96 (18.6) | 0 (0.0) | 0 (0.0) | 328 (100.0) | 0 (0.0) | 0 (0.0) |
| Upper-middle | 246 (15.4) | 116 (15.5) | 130 (15.2) | 95 (17.3) | 83 (17.1) | 40 (13.7) | 28 (10.2) | 32 (11.6) | 73 (15.4) | 44 (14.3) | 95 (18.4) | 0 (0.0) | 0 (0.0) | 0 (0.0) | 246 (100.0) | 0 (0.0) |
| High | 437 (27.3) | 256 (34.3) | 181 (21.1) | 241 (43.8) | 124 (25.5) | 36 (12.4) | 37 (13.2) | 18 (6.3) | 80 (17.0) | 112 (36.4) | 225 (43.5) | 0 (0.0) | 0 (0.0) | 0 (0.0) | 0 (0.0) | 437 (100.0) |
| **Household size**, *χ²*-test |  | χ²(1) = 21.78,  *p* < .001,  *V* = **0.11** | | χ²(3) = 95.81, *p* < .001, *V* = **0.23** | | | | χ²(3) = 18.46, *p* < .001, *V* = **0.10** | | | | χ²(4) = 509.07, *p* < .001, *V* = **0.56** | | | | |
| Single person | 692 (38.6) | 276 (32.8) | 416 (43.6) | 167 (27.2) | 197 (36.6) | 136 (42.2) | 191 (59.6) | 152 (47.7) | 203 (38.8) | 127 (35.1) | 190 (33.9) | 170 (85.1) | 257 (65.7) | 125 (38.2) | 38 (15.5) | 42 (9.6) |
| Several persons | 1102 (61.4) | 564 (67.2) | 538 (56.4) | 445 (72.8) | 341 (63.4) | 186 (57.8) | 129 (40.4) | 166 (52.3) | 320 (61.2) | 235 (64.9) | 371 (66.1) | 30 (14.9) | 134 (34.3) | 202 (61.8) | 208 (84.5) | 395 (90.4) |
| **City size**, *χ²*-test |  | χ²(3) = 1.85,  *p* = .605,  *V* = 0.03 | | χ²(9) = 24.12, *p* = .004, *V* = 0.07 | | | | χ²(9) = 54.58, *p* < .001, *V* = **0.10** | | | | χ²(12) = 10.61, *p* = .563, *V* = 0.05 | | | | |
| < 5,000 | 254 (14.2) | 126 (15.0) | 129 (13.4) | 93 (15.1) | 83 (15.5) | 53 (16.6) | 25 (8.0) | 51 (16.3) | 91 (17.3) | 57 (15.7) | 53 (9.5) | 28 (14.1) | 56 (14.2) | 51 (15.7) | 36 (14.6) | 55 (12.7) |
| 5,000–19,999 | 443 (24.7) | 210 (25.0) | 233 (24.4) | 157 (25.6) | 143 (26.6) | 78 (24.5) | 65 (20.3) | 74 (23.7) | 154 (29.5) | 85 (23.4) | 123 (22.0) | 48 (24.1) | 90 (23.0) | 75 (23.0) | 67 (27.1) | 121 (27.8) |
| 20,000–99,999 | 534 (29.7) | 238 (28.3) | 296 (31.0) | 170 (27.7) | 160 (29.6) | 86 (27.0) | 113 (35.4) | 111 (35.5) | 153 (29.1) | 97 (26.6) | 159 (28.3) | 51 (25.7) | 121 (30.8) | 93 (28.2) | 79 (32.1) | 128 (29.3) |
| ≥ 100,000 | 565 (31.4) | 266 (31.7) | 299 (31.2) | 194 (31.6) | 152 (28.3) | 101 (31.8) | 116 (36.4) | 77 (24.4) | 126 (24.1) | 125 (34.3) | 226 (40.3) | 72 (36.2) | 125 (32.0) | 109 (33.1) | 64 (26.2) | 132 (30.2) |
| **Region**, *χ²*-test |  | χ²(5) = 7.90,  *p* = .162,  *V* = 0.07 | | χ²(15) = 11.54, *p* = .714,  *V* = 0.05 | | | | χ²(15) = 57.41, *p* < .001,  *V* = **0.10** | | | | χ²(20) = 56.49, *p* < .001, *V* = 0.09 | | | | |
| North | 298 (16.5) | 157 (18.6) | 141 (14.8) | 101 (16.5) | 83 (15.4) | 59 (18.3) | 51 (15.9) | 54 (17.1) | 81 (15.4) | 68 (18.8) | 86 (15.3) | 37 (18.8) | 62 (15.8) | 59 (17.9) | 41 (16.7) | 72 (16.4) |
| East | 394 (21.9) | 192 (22.7) | 202 (21.2) | 127 (20.7) | 112 (20.8) | 77 (23.9) | 78 (24.4) | 40 (12.7) | 142 (27.1) | 57 (15.7) | 150 (26.6) | 64 (31.8) | 104 (26.5) | 82 (25.0) | 39 (16.0) | 75 (17.2) |
| Baden-Wuerttemberg | 218 (12.1) | 95 (11.2) | 123 (12.9) | 73 (11.9) | 70 (13.0) | 35 (10.8) | 40 (12.5) | 40 (12.4) | 65 (12.4) | 39 (10.8) | 72 (12.9) | 17 (8.7) | 49 (12.5) | 38 (11.7) | 36 (14.7) | 52 (11.9) |
| Bavaria | 297 (16.5) | 141 (16.7) | 155 (16.3) | 115 (18.8) | 90 (16.8) | 42 (13.1) | 48 (14.9) | 46 (14.6) | 92 (17.5) | 63 (17.3) | 91 (16.3) | 17 (8.6) | 47 (12.0) | 51 (15.5) | 50 (20.4) | 96 (22.1) |
| North Rhine-Westphalia | 370 (20.5) | 162 (19.2) | 208 (21.7) | 118 (19.3) | 111 (20.6) | 69 (21.4) | 71 (22.2) | 81 (25.6) | 87 (16.6) | 92 (25.3) | 100 (17.7) | 34 (17.0) | 85 (21.8) | 59 (17.9) | 54 (22.1) | 79 (18.0) |
| South-West | 224 (12.4) | 98 (11.6) | 126 (13.2) | 78 (12.8) | 73 (13.5) | 40 (12.5) | 33 (10.2) | 56 (17.6) | 58 (11.0) | 44 (12.1) | 64 (11.3) | 30 (15.3) | 45 (11.5) | 39 (11.8) | 25 (10.0) | 63 (14.4) |
| **German federal state**, *χ²*-test |  | χ²(15) = 16.97, *p* = .321,  *V* = 0.10 | | χ²(45) = 69.89, *p* = .010,  *V* = **0.11** | | | | χ²(45) = 118.00, *p* < .001,  *V* = **0.15** | | | | χ²(60) = 99.82, *p* < .001, *V* = **0.13** | | | | |
| Schleswig-Holstein | 80 (4.4) | 44 (5.2) | 36 (3.8) | 33 (5.4) | 17 (3.2) | 14 (4.4) | 15 (4.8) | 22 (7.0) | 23 (4.4) | 15 (4.2) | 19 (3.3) | 8 (4.2) | 19 (4.8) | 17 (5.2) | 11 (4.4) | 17 (3.9) |
| Hamburg | 41 (2.3) | 21 (2.5) | 20 (2.1) | 15 (2.5) | 14 (2.6) | 8 (2.6) | 3 (1.0) | 5 (1.6) | 9 (1.7) | 10 (2.9) | 16 (2.8) | 4 (1.9) | 8 (2.1) | 11 (3.2) | 5 (1.9) | 9 (2.0) |
| Lower Saxony | 164 (9.1) | 81 (9.6) | 83 (8.7) | 49 (7.9) | 51 (9.4) | 29 (9.0) | 31 (9.8) | 27 (8.5) | 47 (9.0) | 35 (9.6) | 48 (8.5) | 20 (10.0) | 33 (8.5) | 29 (8.9) | 23 (9.4) | 43 (9.9) |
| Bremen | 13 (0.7) | 11 (1.3) | 2 (0.2) | 4 (0.7) | 1 (0.2) | 7 (2.2) | 1 (0.2) | 0 (0.0) | 2 (0.4) | 8 (2.2) | 3 (0.6) | 5 (2.6) | 1 (0.3) | 2 (0.6) | 2 (0.9) | 2 (0.5) |
| North Rhine-Westphalia | 370 (20.5) | 162 (19.2) | 208 (21.7) | 118 (19.3) | 111 (20.6) | 69 (21.4) | 71 (22.2) | 81 (25.6) | 87 (16.6) | 92 (25.3) | 100 (17.7) | 34 (17.0) | 85 (21.8) | 59 (17.9) | 54 (22.1) | 79 (18.0) |
| Hesse | 126 (7.0) | 55 (6.5) | 71 (7.5) | 51 (8.4) | 39 (7.3) | 21 (6.5) | 15 (4.6) | 27 (8.3) | 36 (6.9) | 25 (6.9) | 37 (6.6) | 19 (9.7) | 23 (5.9) | 18 (5.5) | 13 (5.4) | 41 (9.4) |
| Rhineland-Palatinate | 86 (4.8) | 37 (4.3) | 49 (5.2) | 24 (3.9) | 31 (5.8) | 16 (4.9) | 15 (4.8) | 28 (8.7) | 16 (3.1) | 17 (4.7) | 24 (4.3) | 11 (5.6) | 21 (5.4) | 16 (4.7) | 11 (4.3) | 18 (4.2) |
| Baden-Wuerttemberg | 218 (12.1) | 95 (11.2) | 123 (12.9) | 73 (11.9) | 70 (13.0) | 35 (10.8) | 40 (12.5) | 40 (12.4) | 65 (12.4) | 39 (10.8) | 72 (12.9) | 17 (8.7) | 49 (12.5) | 38 (11.7) | 36 (14.7) | 52 (11.9) |
| Bavaria | 297 (16.5) | 141 (16.7) | 155 (16.3) | 115 (18.8) | 90 (16.8) | 42 (13.1) | 48 (14.9) | 46 (14.6) | 92 (17.5) | 63 (17.3) | 91 (16.3) | 17 (8.6) | 47 (12.0) | 51 (15.5) | 50 (20.4) | 96 (22.1) |
| Saarland | 11 (0.6) | 6 (0.7) | 5 (0.5) | 3 (0.5) | 2 (0.4) | 3 (1.1) | 3 (0.8) | 2 (0.6) | 5 (1.0) | 2 (0.5) | 2 (0.4) | 0 (0.0) | 1 (0.2) | 5 (1.6) | 1 (0.4) | 4 (0.8) |
| Berlin | 84 (4.6) | 42 (5.0) | 42 (4.4) | 35 (5.7) | 20 (3.7) | 10 (3.0) | 19 (6.0) | 8 (2.6) | 16 (3.1) | 14 (3.7) | 45 (8.0) | 12 (6.2) | 23 (5.8) | 15 (4.7) | 9 (3.8) | 21 (4.8) |
| Brandenburg | 62 (3.4) | 30 (3.6) | 32 (3.3) | 12 (2.0) | 25 (4.7) | 9 (2.7) | 16 (5.0) | 10 (3.2) | 21 (3.9) | 14 (3.8) | 16 (2.9) | 6 (3.1) | 15 (3.9) | 17 (5.2) | 7 (2.7) | 7 (1.7) |
| Mecklenburg-Western Pomerania | 48 (2.7) | 20 (2.3) | 29 (3.0) | 11 (1.8) | 19 (3.6) | 11 (3.4) | 7 (2.1) | 3 (1.0) | 24 (4.6) | 6 (1.6) | 15 (2.7) | 12 (5.8) | 7 (1.7) | 11 (3.4) | 5 (2.2) | 11 (2.5) |
| Saxony | 101 (5.6) | 54 (6.4) | 47 (4.9) | 32 (5.1) | 22 (4.0) | 33 (10.1) | 16 (4.8) | 9 (2.8) | 43 (8.2) | 12 (3.2) | 38 (6.7) | 19 (9.6) | 31 (7.9) | 22 (6.7) | 11 (4.4) | 17 (3.9) |
| Saxony-Anhalt | 50 (2.8) | 24 (2.8) | 27 (2.8) | 17 (2.7) | 13 (2.4) | 8 (2.6) | 12 (3.8) | 4 (1.3) | 22 (4.3) | 6 (1.7) | 14 (2.4) | 6 (2.9) | 15 (3.7) | 9 (2.8) | 5 (1.9) | 6 (1.3) |
| Thuringia | 49 (2.7) | 22 (2.6) | 27 (2.8) | 21 (3.4) | 13 (2.4) | 7 (2.1) | 8 (2.6) | 6 (1.8) | 16 (3.0) | 6 (1.6) | 22 (3.8) | 8 (4.2) | 13 (3.4) | 7 (2.2) | 2 (1.0) | 13 (3.0) |
| **Tobacco consumption**, *χ²*-test |  | χ²(2) = 28.57, *p* < .001, *V* = **0.13** | | χ²(6) = 73.36, *p* < .001, *V* = **0.14** | | | | χ²(6) = 49.88, *p* < .001, *V* = **0.12** | | | | χ²(8) = 28.37, *p* < .001, *V* = 0.09 | | | | |
| Smoker | 342 (19.2) | 186 (22.3) | 156 (16.5) | 152 (25.0) | 121 (22.8) | 47 (14.8) | 18 (5.5) | 90 (28.4) | 112 (21.6) | 67 (18.9) | 65 (11.7) | 50 (26.0) | 80 (20.7) | 55 (16.9) | 38 (15.4) | 62 (14.4) |
| Ex-smoker | 661 (37.1) | 338 (40.6) | 323 (34.1) | 181 (29.8) | 190 (36.0) | 145 (45.3) | 144 (45.1) | 114 (35.9) | 208 (39.9) | 119 (33.7) | 209 (37.5) | 69 (35.6) | 160 (41.2) | 128 (39.4) | 98 (39.7) | 141 (32.8) |
| Non-smoker/occasional | 778 (43.7) | 309 (37.1) | 469 (49.5) | 274 (45.1) | 217 (41.1) | 128 (40.0) | 157 (49.3) | 113 (35.7) | 200 (38.5) | 167 (47.4) | 283 (50.8) | 75 (38.5) | 148 (38.1) | 142 (43.7) | 111 (45.0) | 226 (52.8) |
| **Alcohol consumption**, *χ²*-test |  | χ²(6) = 105.52, *p* < .001,  *V* = **0.24** | | χ²(18) = 87.62, *p* < .001,  *V* = **0.13** | | | | χ²(18) = 74.09, *p* < .001,  *V* = **0.12** | | | | χ²(24) = 104.24, *p* < .001, *V* = **0.13** | | | | |
| Daily | 116 (6.5) | 88 (10.4) | 28 (3.0) | 17 (2.8) | 28 (5.2) | 30 (9.4) | 36 (11.3) | 19 (6.1) | 37 (7.0) | 20 (5.4) | 33 (5.9) | 14 (7.0) | 29 (7.4) | 17 (5.2) | 16 (6.5) | 24 (5.4) |
| Several times a week | 299 (16.7) | 177 (21.0) | 122 (12.9) | 94 (15.3) | 112 (21.1) | 54 (16.6) | 39 (12.3) | 30 (9.6) | 69 (13.1) | 65 (17.9) | 134 (23.9) | 15 (7.7) | 59 (15.1) | 49 (14.8) | 50 (20.3) | 105 (24.0) |
| About once a week | 325 (18.1) | 161 (19.1) | 163 (17.2) | 108 (17.6) | 114 (21.4) | 55 (17.2) | 47 (14.7) | 49 (15.7) | 104 (19.9) | 55 (15.2) | 115 (20.5) | 22 (11.1) | 64 (16.4) | 68 (20.9) | 47 (19.1) | 96 (22.0) |
| Several times a month | 243 (13.6) | 121 (14.3) | 122 (12.9) | 118 (19.2) | 61 (11.5) | 27 (8.4) | 37 (11.7) | 42 (13.3) | 67 (12.8) | 56 (15.5) | 76 (13.5) | 19 (9.3) | 47 (12.0) | 41 (12.6) | 42 (17.1) | 73 (16.6) |
| Once a month | 206 (11.5) | 85 (10.0) | 121 (12.7) | 81 (13.2) | 48 (8.9) | 41 (12.8) | 37 (11.4) | 29 (9.3) | 58 (11.0) | 44 (12.1) | 71 (12.7) | 25 (12.5) | 47 (12.1) | 34 (10.3) | 25 (10.2) | 43 (9.9) |
| Less often | 391 (21.8) | 119 (14.2) | 272 (28.6) | 131 (21.4) | 119 (22.4) | 62 (19.3) | 77 (24.0) | 95 (30.5) | 117 (22.3) | 79 (21.8) | 91 (16.1) | 55 (27.6) | 95 (24.2) | 79 (24.2) | 46 (18.8) | 65 (14.9) |
| Never | 215 (12.0) | 92 (11.0) | 122 (12.8) | 64 (10.5) | 51 (9.5) | 53 (16.4) | 46 (14.5) | 48 (15.5) | 73 (13.9) | 44 (12.1) | 41 (7.4) | 50 (24.8) | 49 (12.6) | 39 (11.9) | 20 (8.1) | 32 (7.2) |

Note: n/a denotes that the χ²-test was not computed. Region summarised the German federal states according to the Nomenclature of Territorial Units for Statistics (NUTS) level 3 by Eurostat (2022) (North = Schleswig-Holstein, Hamburg, Lower Saxony, Bremen; East = Berlin, Brandenburg, Mecklenburg-Western Pomerania, Saxony, Saxony-Anhalt, Thuringia; Baden-Wuerttemberg; Bavaria; North Rhine-Westphalia; South-West = Hesse, Rhineland-Palatinate, Saarland) to enable statistically sound statements about regional tendencies on the basis of a larger sample size. Effect sizes in bold indicate at least small group differences.

### Table S2 Knowledge of diagnostic items of oral cancer by region

| **Sample characteristics** | **The most common sites** | | **Asympto-matic at early stages** | **Majority diagnosis at age 60+** | **Diagnose at advanced stage** | **Appearance of early lesions** | **Sum score diagnostics** |
| --- | --- | --- | --- | --- | --- | --- | --- |
|  | **Tongue** | **Floor of the mouth** |  |  |  |  |  |
|  | % | % | % | % | % | % | *M* (*SD*) |
| **Total** | 58.5 | 33.3 | 37.6 | 37.1 | 64.4 | 36.4 | 2.67 (1.24) |
| **Region,** *overall statistical test* | χ²(5) = 3.86,  *p* = .570,  *V* = 0.05 | χ²(5) = 18.95,  *p* = .002,  *V* = **0.10** | χ²(5) = 15.67,  *p* = .008, *V* = 0.09 | χ²(5) = 8.48,  *p* = .132,  *V* = 0.07 | χ²(5) = 7.24,  *p* = .203,  *V* = 0.06 | χ²(5) = 4.66,  *p* = .458,  *V* = 0.05 | F(5, 1794) = 3.16,  *p* = .008,  *d* = 0.19 |
| North | 59.1 | 31.5 | 32.2 | 36.8 | 68.3 | 41.7 | 2.70 (1.23) |
| East | 54.3 | 30.4 | 32.6 | 35.2 | 60.7 | 36.0 | 2.49 (1.32) |
| Baden-Wuerttemberg | 60.8 | 27.5 | 35.7 | 41.1 | 64.7 | 35.1 | 2.65 (1.27) |
| Bavaria | 58.9 | 43.3 | 42.9 | 42.8 | 63.4 | 34.0 | 2.85 (1.18) |
| North Rhine-Westphalia | 60.1 | 33.4 | 41.7 | 35.1 | 67.9 | 35.1 | 2.73 (1.17) |
| South-West | 60.0 | 33.0 | 41.4 | 32.8 | 61.0 | 36.8 | 2.65 (1.23) |

Note: Effect sizes in bold indicate small group differences. Region summarised the German federal states according to the Nomenclature of Territorial Units for Statistics (NUTS) level 3 by Eurostat (2022) (North = Schleswig-Holstein, Hamburg, Lower Saxony, Bremen; East = Berlin, Brandenburg, Mecklenburg-Western Pomerania, Saxony, Saxony-Anhalt, Thuringia; Baden-Wuerttemberg; Bavaria; North Rhine-Westphalia; South-West = Hesse, Rhineland-Palatinate, Saarland) to enable statistically sound statements about regional tendencies on the basis of a larger sample size. Effect sizes in bold indicate at least small group differences.

### Table S3 Results for post hoc tests on knowledge of diagnostic items of oral cancer by sociodemographic group

| **Sample characteristics** | | **The most common sites: floor of the mouth** | **Asymptom-atic at early stages** | **Majority diagnosis ≤ 60 years** | **Diagnose at advanced stage** | **App-earance of early lesions** | **Sum score diagnostics** |
| --- | --- | --- | --- | --- | --- | --- | --- |
| **Post hoc group 1** | **Post hoc group 2** |  |  |  |  |  |  |
| **Age group,** *overall statistical test* | | n/a | χ²(3) = 52.84,  *p* < .001,  *V* = **0.17** | n/a | χ²(3) = 21.83,  *p* < .001, *V* = **0.11** | χ²(3) = 40.15,  *p* < .001, *V* = **0.15** | F(3, 1790) = 19.31,  *p* < .001, *d* = **0.36** |
| 50–59 | 60–69 | n/a | *p* = 1.000,  *V* = 0.04 | n/a | *p* = 1.000, *V* = 0.00 | *p* = 1.000,  *V* = 0.03 | *p* = 1.000,  *d* = 0.03 |
|  | 70–79 | n/a | *p* < .001,  *V* **= 0.17** | n/a | *p* = .048,  *V* = 0.09 | *p* < .001, *V* **= 0.13** | *p* < .001,  *d* **= 0.39** |
|  | ≥ 80 | n/a | *p* < .001,  *V* **= 0.19** | n/a | *p* = .001,  *V* **= 0.13** | *p* < .001, *V* **= 0.18** | *p* < .001,  *d* **= 0.40** |
| 60–69 | 70–79 | n/a | *p* < .001,  *V* **= 0.14** | n/a | *p* = .066,  *V* = 0.09 | *p* = .013, *V* **= 0.10** | *p* < .001,  *d* **= 0.35** |
|  | ≥ 80 | n/a | *p* < .001,  *V* **= 0.16** | n/a | *p* = .001,  *V* **= 0.13** | *p* < .001, *V* **= 0.16** | *p* < .001,  *d* **= 0.36** |
| 70–79 | ≥ 80 | n/a | *p* = 1.000,  *V* = 0.03 | n/a | *p* = 1.000, *V* = 0.04 | *p* = .809, *V* = 0.06 | *p* = 1.000,  *d* = −0.00 |
| **School education,** *overall statistical test* |  | n/a | χ²(3) = 47.18,  *p* < .001,  *V* = **0.16** | n/a | χ²(3) = 22.26,  *p* < .001,  *V* = **0.11** | n/a | F(3, 1763) = 20.36,  *p* < .001,  *d* = **0.37** |
| Low | Middle | n/a | *p* = 1.000,  *V* = 0.02 | n/a | *p* = 1.000, *V* = 0.07 | n/a | *p* = .648,  *d* = −0.08 |
|  | Advanced | n/a | *p* < .001,  *V* **= 0.16** | n/a | *p* < .001,  *V* **= 0.15** | n/a | *p* < .001,  *d* **=** −**0.46** |
|  | High | n/a | *p* < .001,  *V* **= 0.13** | n/a | *p* < .001,  *V* **= 0.13** | n/a | *p* < .001,  *d* **=** −**0.37** |
| Middle | Advanced | n/a | *p* < .001,  *V* **= 0.18** | n/a | *p* = .078,  *V* = 0.08 | n/a | *p* < .001,  *d* **=** −**0.39** |
|  | High | n/a | *p* < .001,  *V* **= 0.16** | n/a | *p* = .138,  *V* = 0.07 | n/a | *p* < .001,  *d* **=** −**0.30** |
| Advanced | High | n/a | *p* = 1.000,  *V* = 0.02 | n/a | *p* = 1.000, *V* = 0.02 | n/a | *p* = .543,  *d* = -0.04 |
| **Net monthly household income,** *overall statistical test* |  | n/a | χ²(4) = 34.00,  *p* < .001,  *V* = **0.15** | χ²(4) = 18.59,  *p* < .001,  *V* = **0.11** | n/a | n/a | F(4, 1597) = 15.17,  *p* < .001,  *d* = **0.39** |
| Low | Lower-middle | n/a | *p* = 1.000,  *V* = 0.00 | *p* = 1.000, *V* = 0.01 | n/a | n/a | *p* = .313,  *d* = −0.19 |
|  | Middle | n/a | *p* = .329,  *V* = 0.09 | *p* = 1.000, *V* = 0.04 | n/a | n/a | *p* < .001,  *d* **=** −**0.41** |
|  | Upper-middle | n/a | *p* = .042,  *V* **= 0.14** | *p* = .095,  *V* **= 0.12** | n/a | n/a | *p* < .001,  *d* **=** −**0.48** |
|  | High | n/a | *p* = .001,  *V* **= 0.16** | *p* = .125,  *V* = 0.10 | n/a | n/a | *p* < .001,  *d* **=** −**0.54** |
| Lower-middle | Middle | n/a | *p* = .090,  *V* = 0.10 | *p* = 1.000, *V* = 0.05 | n/a | n/a | *p* = .009,  *d* **=** −**0.25** |
|  | Upper-middle | n/a | *p* = .006,  *V* **= 0.14** | *p* = .010,  *V* **= 0.13** | n/a | n/a | *p* < .001,  *d* **=** −**0.33** |
|  | High | n/a | *p* < .001,  *V* **= 0.17** | *p* = .009,  *V* **= 0.12** | n/a | n/a | *p* < .001,  *d* **=** −**0.38** |
| Middle | Upper-middle | n/a | *p* = 1.000,  *V* = 0.04 | *p* = .610,  *V* = 0.08 | n/a | n/a | *p* = 1.000, *d* = −0.07 |
|  | High | n/a | *p* = .317,  *V* = 0.08 | *p* = .878,  *V* = 0.06 | n/a | n/a | *p* = 1.000, *d* = −0.12 |
| Upper-middle | High | n/a | *p* = 1.000,  *V* = 0.03 | *p* = 1.000, *V* = 0.02 | n/a | n/a | *p* = 1.000, *d* = −0.05 |
| **Region,** *overall statistical test* |  | χ²(5) = 18.95,  *p* = .002,  *V* = **0.10** | n/a | n/a | n/a | n/a | n/a |
| North | East | *p* = 1.000,  *V* = 0.01 | n/a | n/a | n/a | n/a | n/a |
|  | Baden-Wuerttem-berg | *p* = 1.000,  *V* = 0.04 | n/a | n/a | n/a | n/a | n/a |
|  | Bavaria | *p* = .043,  *V* **= 0.12** | n/a | n/a | n/a | n/a | n/a |
|  | North Rhine-Westphalia | *p* = 1.000,  *V* = 0.02 | n/a | n/a | n/a | n/a | n/a |
|  | South-West | *p* = 1.000,  *V* = 0.02 | n/a | n/a | n/a | n/a | n/a |
| East | Baden-Wuerttem-berg | *p* = 1.000,  *V* = 0.03 | n/a | n/a | n/a | n/a | n/a |
|  | Bavaria | *p* = .007,  *V* **= 0.13** | n/a | n/a | n/a | n/a | n/a |
|  | North Rhine-Westphalia | *p* = 1.000,  *V* = 0.03 | n/a | n/a | n/a | n/a | n/a |
|  | South-West | *p* = 1.000,  *V* = 0.03 | n/a | n/a | n/a | n/a | n/a |
| Baden-Wuerttem-berg | Bavaria | *p* = .003,  *V* **= 0.16** | n/a | n/a | n/a | n/a | n/a |
|  | North Rhine-Westphalia | *p* = 1.000,  *V* = 0.06 | n/a | n/a | n/a | n/a | n/a |
|  | South-West | *p* = 1.000,  *V* = 0.06 | n/a | n/a | n/a | n/a | n/a |
| Bavaria | North Rhine-Westphalia | *p* = .105,  *V* **= 0.10** | n/a | n/a | n/a | n/a | n/a |
|  | South-West | *p* = .240,  *V* **= 0.11** | n/a | n/a | n/a | n/a | n/a |
| North Rhine-Westphalia | South-West | *p* = 1.000,  *V* = 0.00 | n/a | n/a | n/a | n/a | n/a |

Note: Post hoc tests at the sum score level were calculated using the Bonferroni correction and, in the case of variance heterogeneity, the Games–Howell correction. At the item level, group comparisons were carried out using the χ²-test with Bonferroni correction. Effect sizes in bold indicate small group differences. n/a indicates that no post hoc test was conducted, as post hoc tests were only conducted in the case where the overall group differences showed at least a small effect according to Cohen’s *d*.

*Note:* Region summarised the German federal states according to the Nomenclature of Territorial Units for Statistics (NUTS) level 3 by Eurostat (2022) (North = Schleswig-Holstein, Hamburg, Lower Saxony, Bremen; East = Berlin, Brandenburg, Mecklenburg-Western Pomerania, Saxony, Saxony-Anhalt, Thuringia; Baden-Wuerttemberg; Bavaria; North Rhine-Westphalia; South-West = Hesse, Rhineland-Palatinate, Saarland) to enable statistically sound statements about regional tendencies based on a larger sample size. Effect sizes in bold indicate at least small group differences.

### Table S4 Knowledge of oral cancer risk factors by region

| **Sample characteristics** | **Older age** | **Alcohol consump-tion** | **Tobacco consump-tion** | **Low intake of fruit/ vege-tables** | **Prior oral cancer lesion** | **HPV** | **Sun expo-sure** | **Sum score risk factors** |
| --- | --- | --- | --- | --- | --- | --- | --- | --- |
|  | % | % | % | % | % | % | % | *M (SD)* |
| **Total** | 68.5 | 59.0 | 92.0 | 29.9 | 87.7 | 36.4 | 32.0 | 4.05 (1.38) |
| **Region,** *overall statistical test* | χ²(5) = 10.06,  *p* = .074, *V* = 0.07 | χ²(5) = 8.91,  *p* = .113,  *V* = 0.07 | χ²(5) = 3.50,  *p* = .623,  *V* = 0.04 | χ²(5) = 8.65,  *p* = .124,  *V* = 0.07 | χ²(5) = 3.46,  *p* = .629,  *V* = 0.04 | χ²(5) = 3.87,  *p* = .568,  *V* = 0.05 | χ²(5) = 1.82, *p* = .873,  *V* = 0.03 | F(5, 1794) = 2.06,  *p* = .068,  *d* = 0.16 |
| North | 68.2 | 61.0 | 92.3 | 30.8 | 88.8 | 37.0 | 32.0 | 4.10 (1.31) |
| East | 67.6 | 54.9 | 90.0 | 27.7 | 85.5 | 34.1 | 30.2 | 3.90 (1.36) |
| Baden-Wuerttemberg | 63.3 | 63.7 | 93.5 | 26.5 | 89.3 | 38.9 | 34.0 | 4.09 (1.38) |
| Bavaria | 75.4 | 58.0 | 93.2 | 31.5 | 88.7 | 38.8 | 30.4 | 4.16 (1.39) |
| North Rhine-Westphalia | 66.7 | 62.3 | 92.3 | 34.6 | 88.2 | 37.0 | 32.8 | 4.14 (1.40) |
| South-West | 69.1 | 54.9 | 91.3 | 25.6 | 86.2 | 32.6 | 34.1 | 3.94 (1.42) |

*Note:* Effect sizes in bold indicate small group differences. Region summarised the German federal states according to the Nomenclature of Territorial Units for Statistics (NUTS) (North = Schleswig-Holstein, Hamburg, Lower Saxony, Bremen; East = Berlin, Brandenburg, Mecklenburg-Western Pomerania, Saxony, Saxony-Anhalt, Thuringia; Baden-Wuerttemberg; Bavaria; North Rhine-Westphalia; South-West = Hesse, Rhineland-Palatinate, Saarland) level 3 by Eurostat (2022) to enable statistically sound statements about regional tendencies based on a larger sample size. Effect sizes in bold indicate at least small group differences.

### Table S5 Results for post hoc tests on knowledge of oral cancer risk factors by

### sociodemographic group

| **Sample characteristics** | | **Older age** | **Tobacco consumpt-ion** | **Prior oral cancer lesion** | **HPV** | **Sum score risk factors** |
| --- | --- | --- | --- | --- | --- | --- |
| **Post hoc group 1** | **Post hoc group 2** |  |  |  |  |  |
| **Age group,** *overall statistical test* |  | χ²(3) = 28.69, *p* < .001,  *V* = **0.13** | n/a | χ²(3) = 45.89, *p* < .001,  *V* = **0.16** | n/a | Welch F (3, 842.18) = 11.06,  *p* < .001,  *d* = **0.28** |
| 50–59 | 60–69 | *p* = .294,  *V* = 0.06 | n/a | *p* = 1.000,  *V* = 0.03 | n/a | *p* = .856,  *d* = 0.05 |
|  | 70–79 | *p* < .001,  *V* = **0.15** | n/a | *p* < .001,  *V* = **0.17** | n/a | *p* < .001,  *d* = **0.39** |
|  | ≥ 80 | *p* < .001,  *V* = **0.14** | n/a | *p* < .001,  *V* = **0.18** | n/a | *p* < .001,  *d* = **0.39** |
| 60–69 | 70–79 | *p* = .035,  *V* = 0.09 | n/a | *p* = .001,  ***V* = 0.13** | n/a | *p* = .001,  *d* = **0.27** |
|  | ≥ 80 | *p* = .782,  *V* = 0.08 | n/a | *p* < .001,  *V* = **0.15** | n/a | *p* = .002,  *d* = **0.27** |
| 70–79 | ≥ 80 | *p* = 1.000,  *V* = 0.01 | n/a | *p* = 1.000,  *V* = 0.02 | n/a | *p* = .999,  *d* = 0.01 |
| **School education,** *overall statistical test* |  | χ²(3) = 51.51, *p* < .001,  *V* = **0.17** | χ²(3) = 18.36, *p* < .001,  *V* = **0.10** | χ²(3) = 39.66, *p* < .001,  *V* = **0.15** | χ²(3) = 20.36, *p* < .001,  *V* = **0.11** | Welch F (3, 877.63) = 18.19,  *p* < .001,  *d* = **0.36** |
| Low | Middle | *p* = 1.000,  *V* = 0.03 | *p* = 1.000,  *V* = 0.04 | *p* = .001,  *V* = **0.13** | *p* = .046,  *V* = 0.09 | *p* = .002,  *d* = −**0.26** |
|  | Advanced | *p* < .001,  *V* = **0.15** | *p* = .019,  *V* = **0.11** | *p* < .001,  *V* = **0.19** | *p* = .005,  ***V* = 0.13** | *p* < .001,  *d* = −**0.43** |
|  | High | *p* < .001,  *V* = **0.20** | *p* = .001,  *V* = **0.13** | *p* < .001,  *V* = **0.19** | *p* < .001,  ***V* = 0.15** | *p* < .001,  *d* = −**0.51** |
| Middle | Advanced | *p* = .002,  *V* = **0.12** | *p* = .256,  *V* = 0.07 | *p* = .491,  *V* = 0.06 | *p* = 1.000,  *V* = 0.03 | *p* = .065,  *d* = −0.17 |
|  | High | *p* < .001,  *V* = **0.18** | *p* = .038,  *V* = 0.08 | *p* = .218,  *V* = 0.06 | *p* = .248,  *V* = 0.06 | *p* < .001,  *d* = −**0.23** |
| Advanced | High | *p* = .786,  *V* = 0.05 | *p* = 1.000,  *V* = 0.01 | *p* = 1.000,  *V* = 0.00 | *p* = 1.000,  *V* = 0.03 | *p* = .757,  *d* = -0.20 |
| **Net monthly household income,** *overall statistical test* |  | χ²(4) = 22.30, *p* < .001,  *V* = **0.12** | χ²(4) = 24.12, *p* < .001,  *V* = **0.12** | χ²(4) = 24.95, *p* < .001,  *V* = **0.12** | n/a | Welch F (4, 691.18) = 6.67,  *p* < .001,  *d* = **0.26** |
| Low | Lower-middle | *p* = 1.000,  *V* = 0.02 | *p* = 1.000,  *V* = 0.05 | *p* = .088,  *V* = **0.11** | n/a | *p* = .420,  *d* = −0.16 |
|  | Middle | *p* = 1.000,  *V* = 0.04 | *p* = .192,  *V* = **0.10** | *p* = .044,  *V* = **0.12** | n/a | *p* = .726,  *d* = −0.12 |
|  | Upper-middle | *p* = 1.000,  *V* = 0.06 | *p* = .193,  *V* = **0.11** | *p* < .001,  *V* = **0.21** | n/a | *p* = .020,  *d* = −**0.30** |
|  | High | *p* = .009,  ***V* = 0.13** | *p* < .001,  *V* = **0.19** | *p* = .001,  *V* = **0.16** | n/a | *p* < .001,  *d* = −**0.38** |
| Lower-middle | Middle | *p* = 1.000,  *V* = 0.05 | *p* = 1.000,  *V* = 0.05 | *p* = 1.000,  *V* = 0.02 | n/a | *p* = .975,  *d* = 0.04 |
|  | Upper-middle | *p* = .476,  *V* = 0.08 | *p* = 1.000,  *V* = 0.05 | *p* = .183,  *V* = 0.09 | n/a | *p* = .361,  *d* = −0.14 |
|  | High | *p* < .001,  *V* = **0.15** | *p* = .001,  *V* = **0.13** | *p* = 1.000,  *V* = 0.05 | n/a | *p* = .016,  *d* = −**0.22** |
| Middle | Upper-middle | *p* = 1.000,  *V* = 0.03 | *p* = 1.000,  *V* = 0.01 | *p* = .577,  *V* = 0.08 | n/a | *p* = .134,*d* = −0.20 |
|  | High | *p* = .055,  *V* = **0.10** | *p* = .152,  *V* = 0.09 | *p* = 1.000, *V* = 0.04 | n/a | *p* = .002, *d* = −**0.27** |
| Upper-middle | High | *p* = .623,  *V* = 0.07 | *p* = .389,  *V* = 0.08 | *p* = 1.000,  *V* = 0.04 | n/a | *p* = .884,  *d* = −0.07 |
| **Alcohol consumption,** *overall statistical test* |  | n/a | n/a | χ²(6) = 31.46, *p* < .001,  *V* = **0.13** | n/a | n/a |
| Daily | Several times a week | n/a | n/a | *p* = .079,  *V* = **0.14** | n/a | n/a |
|  | About once a week | n/a | n/a | *p* = .013,  *V* = **0.16** | n/a | n/a |
|  | Several times a month | n/a | n/a | *p* < .001,  *V* = **0.24** | n/a | n/a |
|  | Once a month | n/a | n/a | *p* = 1.000,  *V* = 0.07 | n/a | n/a |
|  | Less often | n/a | n/a | *p* = 1.000,  *V* = 0.06 | n/a | n/a |
|  | Never | n/a | n/a | *p* = 1.000,  *V* = 0.05 | n/a | n/a |
| Several times a week | About once a week | n/a | n/a | *p* = 1.000,  *V* = 0.02 | n/a | n/a |
|  | Several times a month | n/a | n/a | *p* = .941,  *V* = 0.09 | n/a | n/a |
|  | Once a month | n/a | n/a | *p* = 1.000,  *V* = 0.08 | n/a | n/a |
|  | Less often | n/a | n/a | *p* = 1.000,  *V* = 0.07 | n/a | n/a |
|  | Never | n/a | n/a | *p* = .494,  *V* = 0.10 | n/a | n/a |
| About once a week | Several times a month | n/a | n/a | *p* = 1.000,  *V* = 0.07 | n/a | n/a |
|  | Once a month | n/a | n/a | *p* = .626,  *V* = 0.09 | n/a | n/a |
|  | Less often | n/a | n/a | *p* = .259,  *V* = 0.09 | n/a | n/a |
|  | Never | n/a | n/a | *p* = .123,  *V* = **0.12** | n/a | n/a |
| Several times a month | Once a month | n/a | n/a | *p* = .015,  *V* = **0.16** | n/a | n/a |
|  | Less often | n/a | n/a | *p* = .005,  *V* = **0.15** | n/a | n/a |
|  | Never | n/a | n/a | *p* = .002,  *V* = **0.19** | n/a | n/a |
| Once a month | Less often | n/a | n/a | *p* = 1.000,  *V* = 0.00 | n/a | n/a |
|  | Never | n/a | n/a | *p* = 1.000,  *V* = 0.02 | n/a | n/a |
| Less often | Never | n/a | n/a | *p* = 1.000,  *V* = 0.02 | n/a | n/a |

*Note:* Post hoc tests at the sum score level were calculated using the Bonferroni correction and, in the case of variance heterogeneity, the Games–Howell correction. At the item level, group comparisons were carried out using the χ²-test with Bonferroni correction. Effect sizes in bold indicate small group differences. n/a indicates that no post hoc test was conducted, as post hoc tests were only conducted in the case where the overall group differences showed at least a small effect according to Cohen’s *d*.

### References

Eurostat (2022). Statistical regions in the European Union and partner countries: NUTS and statistical regions 2021 - 2022 edition. Publications Office of the European Union. <https://ec.europa.eu/eurostat/documents/3859598/15193590/KS-GQ-22-010-EN-N.pdf>
